# Impact of future climate trend and fluctuation on winter wheat yield in the North China Plain and adaptation strategies

**DOI:** 10.1038/s41598-025-06370-6

**Published:** 2025-07-01

**Authors:** Jinpeng Hu, Yichen Li, Peijun Shi

**Affiliations:** 1 State Key Laboratory of Earth Surface Processes and Disaster Risk Reduction (ESPDRR), Beijing, 100875 China; 2 Key Laboratory of Environmental Change and Natural Disasters of Chinese Ministry of Education, Beijing Normal University, Beijing, 100875 China; 3 College of Arts and Sciences, Beijing Normal University, Zhuhai, 519087 China; 4 College of Safety and Environmental Engineering, Shandong University of Science and Technology, Qingdao, 266590 China

**Keywords:** Climate drivers, Crop model simulation, Irrigation and rain-fed systems, Adaptive management, Climate sciences, Natural hazards

## Abstract

Research on the impacts of climate change on crop yield is crucial for improving agricultural management practices and enhancing climate adaptability. Although previous studies have explored the effects of climate trends and fluctuations on wheat yield, their combined impacts under future climate scenarios in the North China Plain (NCP) remain insufficiently understood. This study employs the DSSAT model to analyze the impacts of future climate trends and fluctuations on winter wheat yield. The results indicate that in the 2030s, the benefits of increased precipitation outweighed the losses from rising temperatures, leading to a 1.5% increase in winter wheat yield in the NCP. However, by the 2080s, continuous temperature rise dominated yield reduction, resulting in a 13.4% decline, which exceeded the compensatory capacity of increased precipitation. Irrigated wheat was primarily influenced by temperature trends, while rainfed systems were more sensitive to precipitation fluctuations. Delaying the planting date and increasing field fertility could mitigate 6–7.5% of the potential losses caused by rising temperatures, whereas increasing irrigation had limited mitigation effects (only improving yield by 3%). This study quantifies the climate impact benefits on winter wheat in the NCP and highlights the need for prioritizing heat-tolerant varieties and optimizing sowing and fertilization practices over water-intensive adaptation strategies. The findings provide decision-making support for ensuring food security under a warming climate.

**Supplementary Information:**

The online version contains supplementary material available at 10.1038/s41598-025-06370-6.

## Introduction

Climate change has significantly impacted crop growth and productivity, with agriculture being highly vulnerable to its effects^1–3^. Studies indicate that global agricultural production demand is expected to increase by 30–62% by 2050^4^, while the current growth rate of food production lags far behind the growth rate of food demand^5,6^. Over the past 30 years, climate change has accounted for approximately 32–39% of crop yield variability^7^, and global warming is projected to reduce crop yields in the coming decades^8,9^. The North China Plain (NCP), one of China’s most important grain-producing regions, accounts for over 50% of the country’s winter wheat planting area and plays a pivotal role in ensuring national food security^10^. However, in recent years, significant climate change trends in the NCP, including rising average temperatures, shifting precipitation patterns, and frequent extreme weather events, have posed severe challenges to wheat production^11,12^.

As temperatures rise in the NCP, the frequency and intensity of extreme climate events have increased^13^. Future climate projections suggest that the region may face more pronounced warming trends, greater precipitation variability, and changes in solar radiation, all of which could affect crop phenology, growth cycles, and final yields^14^. Specifically, rising temperatures may accelerate wheat growth stages, with high temperatures particularly impacting grain filling and seed setting. On the other hand, increased precipitation variability may significantly affect rainfed wheat^15–17^. The uncertainty of future climate projections highlights the importance of comprehensively assessing the impacts of climate change on crop production^18–20^.

The agricultural impacts of climate change are multidimensional. According to the Intergovernmental Panel on Climate Change (IPCC) Sixth Assessment Report (AR6)^21^, climate change impacts agriculture through both long-term climate trends and short-term climate variability. Climate trends refer to directional changes in long-term climate, such as global warming-induced temperature increases, which are typically stable and persistent. In contrast, climate variability refers to internal variability of the climate system, such as interannual or monthly fluctuations in temperature and precipitation, which are more short-term and uncertain^22^. The IPCC AR6 emphasizes that agriculture is one of the sectors most vulnerable to these two types of climate change^21^. Climate trends can gradually alter the suitability of cropping systems, while short-term climate variability increases the frequency and severity of extreme weather events, posing immediate and severe risks to agricultural production. Although research on climate trends and variability has increased in recent years^23–26^, systematic evaluations of their combined effects on agricultural production in the NCP remain limited^27–30^. Therefore, assessing the combined effects of these climate factors is crucial for comprehensively understanding agricultural vulnerability and informing effective adaptation strategies.

Addressing the threat of climate change to wheat production requires the development of effective adaptation strategies. Studies have demonstrated that measures such as adjusting sowing dates, optimizing water and nutrient management, and breeding heat-tolerant cultivars can significantly mitigate the adverse impacts of climate change^31^. For instance, delayed sowing can avoid autumn heat stress on seedling growth^32^, while enhanced soil fertility alleviates the negative effects of high temperatures^33^. A systematic assessment of multiple strategies in the North China Plain, particularly research on the differential response mechanisms between irrigated and rainfed systems, is critical for regional agricultural production to adapt to future climate change.

This study utilizes the DSSAT-CERES-Wheat model combined with CMIP6 climate data to systematically evaluate the impacts of future climate change on winter wheat in the NCP. The DSSAT-CERES-Wheat model has been widely used for global wheat yield simulations and demonstrates strong applicability to Chinese wheat regions^30,34^. Compared to other crop models, DSSAT-CERES-Wheat has been extensively studied for simulating complex management practices in the NCP, better reflecting regional agricultural practices^35,36^. Additionally, CMIP6 climate data provide higher resolution and richer climate scenarios, enabling a more detailed assessment of future climate change impacts and reducing systematic errors^21^.

This study aims to quantify the impacts of climate trends and variability on winter wheat yield, assess their relative contributions, reveal the differential responses of irrigated and rainfed winter wheat to climate change, identify the most sensitive meteorological factors, and evaluate the effectiveness of adaptation strategies (e.g., adjusting planting dates, optimizing fertilization, and irrigation management) in mitigating climate change impacts. By integrating the DSSAT-CERES-Wheat model, CMIP6 climate data, and agricultural meteorological station data, this study provides a scientific basis for regional agricultural resource management and climate change adaptation policies.

## Materials and methods

### Study area

The North China Plain is the largest wheat-producing region in China^37^. The yield of wheat in this region account for over 60% of the total wheat in China, which is strategically important for the country’s food security^38^. As the majority of wheat planting areas in the NCP are located in Hebei, Shandong, and Henan provinces, considering factors such as regional scope, administrative divisions, and consistency in cropping systems, this study focused on these three provinces as research areas for the NCP. The geographic location of the study area is 31°–43° N, 110°–123° E, and the region is dominated by a warm-temperate monsoon climate characterized by rainfall and high temperatures in the same season. Most of the NCP has a winter wheat-summer maize crop rotation, with irrigated agriculture in the northern part of the NCP and rainfed agriculture in the southern part.

**Fig. 1:**
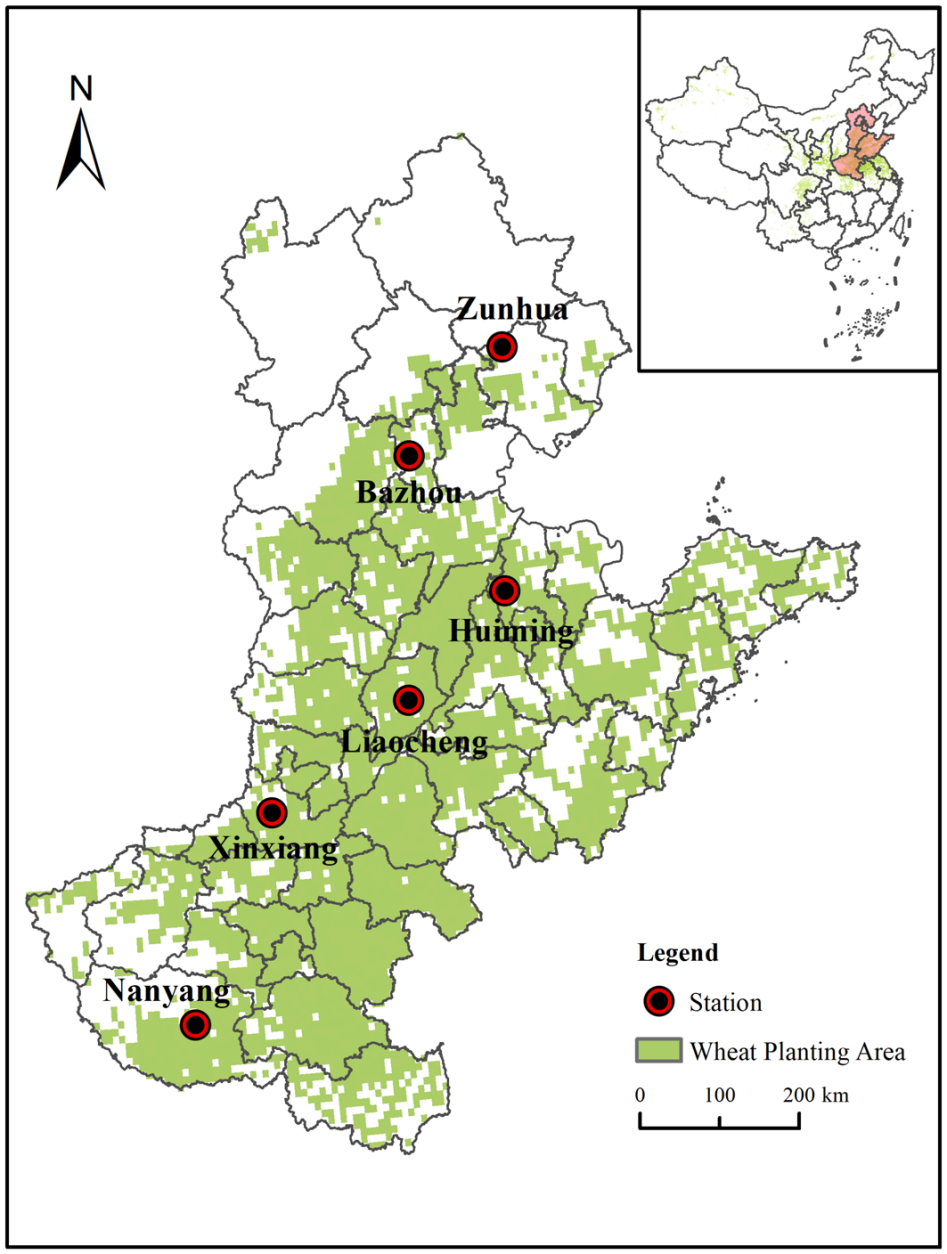
Study area and agricultural meteorological stations. (*Note*: The map was generated using ArcMap 10.6 software, URL: https://www.arcgis.com/index.html, by authors).

This study selected six agrometeorological stations in the NCP, including Zunhua and Bazhou stations in Hebei Province, Huimin and Liaocheng stations in Shandong Province, and Nanyang and Xinxiang stations in Henan Province (Fig. 1). These stations cover different climatic zones and agricultural practices within the NCP, with Nanyang station representing rainfed agriculture and the remaining stations representing irrigated agriculture. The selection of these stations ensures a comprehensive reflection of climate change and agricultural production systems within the study area. Geographically, the stations are evenly distributed across most regions of the NCP, and their climatic gradients align with the spatial distribution characteristics of temperature, precipitation, and solar radiation across the NCP, effectively representing the climatic and geographical diversity of the region (Fig. S1).

Each station varies in climatic conditions, soil types, and management practices (e.g., irrigation and fertilization schemes, planting dates), which enables the reflection of the differential impacts of climate change on agricultural production within the region. By using the DSSAT-CERES-Wheat model, localized climate, soil, and management practice models can be constructed for each station, better capturing regional heterogeneity^39^. Through a comprehensive analysis of the simulation results from these six stations, this study effectively assesses the potential impacts of climate change on winter wheat yield across the entire NCP, providing meaningful references and predictions for broader regions.

### Research data

The historical meteorological data used in this study were obtained from the China Meteorological Data Service Center (http://data.cma.cn), covering daily precipitation, sunshine duration, maximum temperature, and minimum temperature from 1987 to 2014. Daily solar radiation (SR) was derived using the empirical Angstrom function^40^ to convert sunshine duration into SR. Soil data for the crop model were sourced from the China Soil Database (http://vdb3.soil.csdb.cn) and the Harmonized World Soil Database, including soil layer depth, bulk density, soil texture properties (wilting point, saturated water content, field capacity), root growth coefficient, drainage coefficient, and runoff coefficient (Table S1).

The field management data required for the crop model were provided by the National Meteorological Information Center of China (1981–2012), including crop varieties, sowing quantity, sowing and harvest dates, fertilization quantity, and irrigation practices. These data were primarily used for model calibration, validation, and initial condition setup. The parameters of the DSSAT model (e.g., photoperiod sensitivity P1D, grain filling coefficient P5) were calibrated using multi-year observational data (e.g., yield, growth stages) to ensure the accuracy of the simulation results^41^. Additionally, the average values of multi-year observational data were used as initial conditions for model runs (e.g., planting date, fertilization, and irrigation) to reduce the impact of randomness in single-year data and ensure the stability of the simulation results^42^. For future scenarios, field management practices were assumed to remain at current levels, without considering the effects of cultivar replacement. The specific initial values for model inputs were adjusted based on station records and cultivar characteristics (Table S2).

Future climate data were based on five global climate models (GCMs) from CMIP6: GFDL-ESM4, IPSL-CM6A-LR, MPI-ESM1-2-LR, MRI-ESM2-0, and UKESM1-0-LL. These GCMs are commonly used to study the impacts of climate change on crops and provide comprehensive coverage of spatial variations in mean temperature and relative precipitation at regional and global scales^43,44^. The data were sourced from the NASA Global Daily Downscaled Projections (NEX-GDDP-CMIP6), a high-resolution (0.25° × 0.25°) downscaled daily dataset generated from the Scenario Model Intercomparison Project (ScenarioMIP) within the Coupled Model Intercomparison Project Phase 6 (CMIP6). Three emission scenarios—low (SSP1-2.6), medium (SSP2-4.5), and high (SSP5-8.5)—were selected for this study. The dataset includes daily maximum temperature, minimum temperature, precipitation, and solar radiation data from 2015 to 2100. Compared to previous climate models, CMIP6 exhibits higher stability and applicability in simulating climate change, making it more suitable for regional agricultural impact assessments in the NCP^45–47^.

Due to potential systematic biases in CMIP6 climate model data at the regional scale, which may arise from insufficient model resolution, imperfect parameterization of physical processes, and incomplete representation of regional climate characteristics^48^, this study employed the Delta method for bias correction to improve the regional applicability of the data^49,50^. The method used historical observational meteorological data from the six stations as a reference to correct the model output data for the corresponding grid cells. By calculating the average bias (e.g., temperature, precipitation, solar radiation) between historical observational data (1987–2014) and CMIP6 model output data for the same period, and applying this bias to future scenario data (2015–2098), systematic errors were reduced. The corrected data significantly decreased the root mean square error (RMSE) between future projected data and observational data (Table S3), improving data consistency and providing reliable climate inputs for subsequent crop model simulations. Detailed steps of the correction method are provided in Fig. S2. By introducing bias correction and using multi-model ensemble averaging, this study minimized limitations and biases caused by data resolution and heterogeneity. These measures enhanced data reliability and simulation accuracy, providing a stable foundation for further research to better reflect regional climate change characteristics.

### Research methods

#### DSSAT crop model validation and simulation

The DSSAT model can simulate crop growth and development daily and can respond to multiple factors, including crop genetic characteristics, management practices, environment, nitrogen, and water stress, and has been widely used internationally^39^. Calibration and validation of six stations in the North China Plain were conducted using the data records of the agricultural gas stations of the China Meteorological Administration. Many well-established research methods have been used to calibrate and simulate the model parameters^51^. For each site, the model was calibrated using a generalized likelihood uncertainty estimation (GLUE) for multiple years of observations of the same variety, and the observations from the remaining years were used for validation (Table S4). The validation performance was assessed by observing and simulating the predicted deviation (PD), standard root-mean-square error (NRMSE), and R^2^ between the yield, flowering date, and maturity date as follows:

1 $$PD=\frac{{S}_{i}-{O}_{i}}{{O}_{i}}$$

2 $$RMSE=\sqrt{\frac{\sum_{i=1}^{n}{\left({S}_{i}-{O}_{i}\right)}^{2}}{n}}$$

3 $$NRMSE=\frac{\text{RMSE}}{\text{O}}*100\text{\%}$$

4 $${R}^{2}=\frac{{\left[\sum_{i=1}^{n}{(O}_{i}-O)({S}_{i}-S)\right]}^{2}}{\sum_{i=1}^{n}{\left({O}_{i}-O\right)}^{2}\sum_{i=1}^{n}{\left({S}_{i}-S\right)}^{2}}$$

where, *O*_*i*_ is the observed value, *S*_*i*_ is the simulated value, *O* is the measured average value, *S* is the simulated average value, and *n* is the number of samples. In general studies, if the calculated result of *PD* is within ± 15%, the error can be considered to be within the acceptable range, and the model is verified. When *NRMSE* < 10%, the simulation effect of the model was considered to be excellent. The closer the correlation coefficient *R*^*2*^ is to 1, the better the validation performance of the model^52^.

#### Climate change scenario setting

**Fig. 2:**
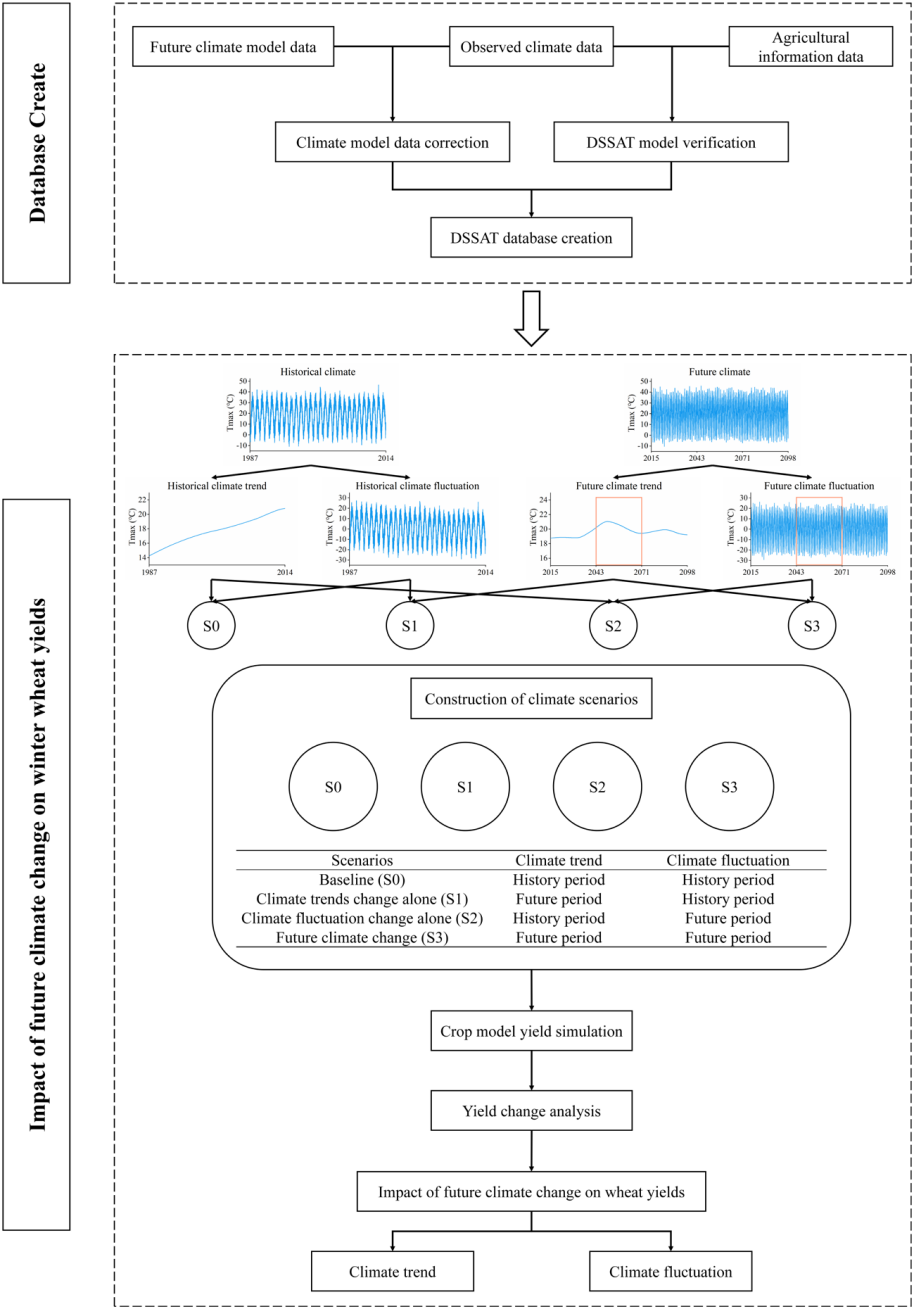
Evaluation framework for the impact of climate change scenarios on winter wheat yield.

To evaluate the impacts of climate change on crop yield and elucidate the contributions of climate trends and fluctuations, this study constructed a baseline scenario (S0) and three climate change scenarios (S1, S2, and S3) (Fig. 2). The future period was divided into three subperiods for comparative analyses: the near-term (2030s: 2015–2042), mid-term (2050s: 2043–2070), and far-term (2080s: 2071–2098). The baseline scenario (S0) used historical climate data from 1987 to 2014, retaining the original climate trends and fluctuations as a reference for comparison. In this study, future climate scenarios were simulated using a fixed historical CO_2_ concentration of 380 ppm(the historical average level). By maintaining a fixed CO_2_ concentration, this study focuses on the independent impacts of climate variables, providing a clearer understanding of the roles of temperature, precipitation, and radiation.

The future climate change scenarios included the comprehensive scenario (S3), trend scenario (S1), and fluctuation scenario (S2). The comprehensive scenario (S3) reflects the combined changes in future climate trends and fluctuations, using bias-corrected CMIP6 data from 2015 to 2098. The trend scenario (S1) considers only changes in climate trends, generated by superimposing future climate trends on historical climate fluctuations. Climate trends were extracted using the residual term from Empirical Mode Decomposition (EMD), reflecting long-term changes^53^. The fluctuation scenario (S2) considers only changes in climate fluctuations, generated by superimposing future climate fluctuations on historical climate trends. Climate fluctuations were extracted using the Intrinsic Mode Functions (IMFs) from EMD, reflecting interannual variability.

The scenario construction employed Empirical Mode Decomposition (EMD), a non-stationary method widely used in meteorological analysis for time-series decomposition^53^. By decomposing temperature, precipitation, and radiation, the time series was divided into multiple IMFs and a residual term. The residual term represents climate trends, while the IMFs represent climate fluctuations. By separating and recombining trends and fluctuations, the S1, S2, and S3 scenario data were generated. Specifically, the residual term from future climate data was extracted using EMD to represent climate trends, and the IMFs were extracted to represent climate fluctuations. Finally, scenario data were generated by combining trends and fluctuations: S1 scenario combines future climate trends with historical climate fluctuations, S2 scenario combines historical climate trends with future climate fluctuations, and S3 scenario combines future climate trends with future climate fluctuations. This method enables the quantification of the independent and interactive impacts of climate trends and fluctuations on winter wheat yield, providing a scientific basis for developing adaptation strategies.

#### Evaluation of the impact of climate trend and fluctuation on winter wheat yield in the North China Plain

Four scenarios (S0, S1, S2, and S3) were constructed for three climatic factors: temperature, precipitation, and radiation. The calibrated DSSAT model was used to simulate the near-term (2015–2042), mid-term (2043–2070) and far-term (2071–2098) wheat yields under SSP126, SSP245, and SSP585. The yield change is obtained by calculating the yield difference (${\Delta }_{i,t}^{c}$) between the simulated yield and the simulated yield in the historical base period. In addition to considering the effects of climate mean and fluctuation, the interaction between the two represents the alteration in the relevant yield under the joint influence of changes in climate mean and fluctuation, minus the total of alterations in the relevant yield under the influence of changes in climate trend or fluctuation individually (${\Delta }_{int,t}^{c}$), The contribution rate is obtained by calculating the percentage change in yield for a specific climate change component relative to the total climate change in yield for the base period^54,55^.

5 $${\Delta }_{i,t}^{c}=\frac{{Y}_{i,t}^{c}-{Y}_{0}^{c}}{{Y}_{0}^{c}}\times 100\%$$

6 $${\Delta }_{int,t}^{c}={\Delta }_{3,t}^{c}-\left({\Delta }_{1,t}^{c}+{\Delta }_{2,t}^{c}\right)$$

7 $${R}_{k,t}^{c}=\frac{{\Delta }_{k,t}^{c}}{\left|{\Delta }_{m,t}^{c}\right|+\left|{\Delta }_{v,t}^{c}\right|+\left|{\Delta }_{int,t}^{c}\right|}$$

where c = pr, sr, temp represent precipitation, solar radiation, and temperature, respectively; i = 1,2,3 denotes the scenarios involving changes in climate components; i = 0 represents the historical baseline scenario; and t = the 2030s, 2050s, and the 2080s.

#### Exploration of climate adaptation strategies

Wheat yield is influenced not only by climatic factors but also by anthropogenic factors, with technological advancements and field management practices being the primary factors directly affecting wheat production processes^56^. To ensure high crop yields and mitigate the adverse effects of climate change, adaptive agricultural management practices are necessary. Common measures include variety updates, adjusting planting dates, and improving fertilization and irrigation conditions^57–59^. Based on the response mechanism of crop yield to climate change, this study explores the adoption of adaptive field management measures to alleviate and eliminate the potential adverse impacts of future climate change, using agricultural sites in the NCP that are significantly affected by yield reductions as examples.

Combining the feasibility of regional agricultural technological advancements and irrigation system improvements, the model was set with three different levels of agricultural management practices (compared to the original level: irrigation water increased by 25%, 50%, and 75%; fertilization increased by 25%, 50%, and 75%; and planting dates delayed by 5 days, 10 days, and 15 days, with the original baseline level being the multi-year average of data recorded at the agricultural meteorological stations Table S2) (Table 1). These were compared with the original management level under the future climate change scenario (S3) to investigate the mitigating effects of irrigation, fertilization, and adjusting planting dates on yield reduction caused by future climate change.

**Table 1:** Design options for adaptation measures.

Adaptation measure	Management level		
Irrigate	+ 25%	+ 50%	+ 75%
Fertilize	+ 25%	+ 50%	+ 75%
Planting date	+ 5 days	+ 10 days	+ 15 days

To ensure the feasibility of the proposed measures, this study conducted a carrying capacity assessment based on historical water resources and fertilization data for cultivated land in the NCP (Fig. S3). In recent years, water resources for cultivated land in the NCP have shown a declining trend, with overall water resource carrying capacity remaining limited, while the application of agricultural fertilizers has increased annually. By comparing historical water resource utilization and fertilizer application, the current irrigation improvement levels (+ 25%, + 50%, + 75%) and fertilization schemes do not exceed the carrying capacity of regional resources, ensuring the rationality and feasibility of the proposed adaptation measures. Furthermore, future scenario analysis will further validate the effectiveness of these improvement measures in addressing climate change.

## Results

### Crop model validation and applicability assessment

After calibration and validation, the DSSAT crop model with adjusted parameters effectively captured the flowering date, maturity date, and yield of winter wheat (Table S5; Fig. S4). The R^2^ values for the flowering stage, maturity stage, and wheat yield were 0.71, 0.79, and 0.81, respectively, with PD values of less than 15% and NRMSE values of 3.8%, 2.7%, and 5.7% (Table 2), indicating high consistency between simulated and observed values^52^. Additionally, the analysis of the coefficient of variation (CV) for yield across stations (Table S6) showed a range of 9.9–16.2%, reflecting the sensitivity of yield variability to climate within the region. Overall, the model’s ability to represent key crop growth parameters validates its applicability in the North China Plain.

**Table 2:** Model calibration and validation assessment.

	Days to flowering from planting	Days to maturity from planting	Yield
*PD*	3.0%	2.1%	4.4%
*R*^*2*^	0.71	0.79	0.81
*NRMSE*	3.8%	2.7%	5.7%

This study primarily focuses on the simulation of winter wheat growth stages and final yield, as these indicators are core parameters for evaluating crop model performance and have significant practical implications in agricultural production^39^. While other crop parameters, such as biomass, are also crucial for model calibration and evaluation, detailed analysis of these parameters was not conducted due to limitations in obtaining measured data. However, the parameters validated in this study are sufficient to meet the basic assessment of model performance.

### Changes of climate trend and fluctuation in future winter wheat growth period

**Fig. 3:**
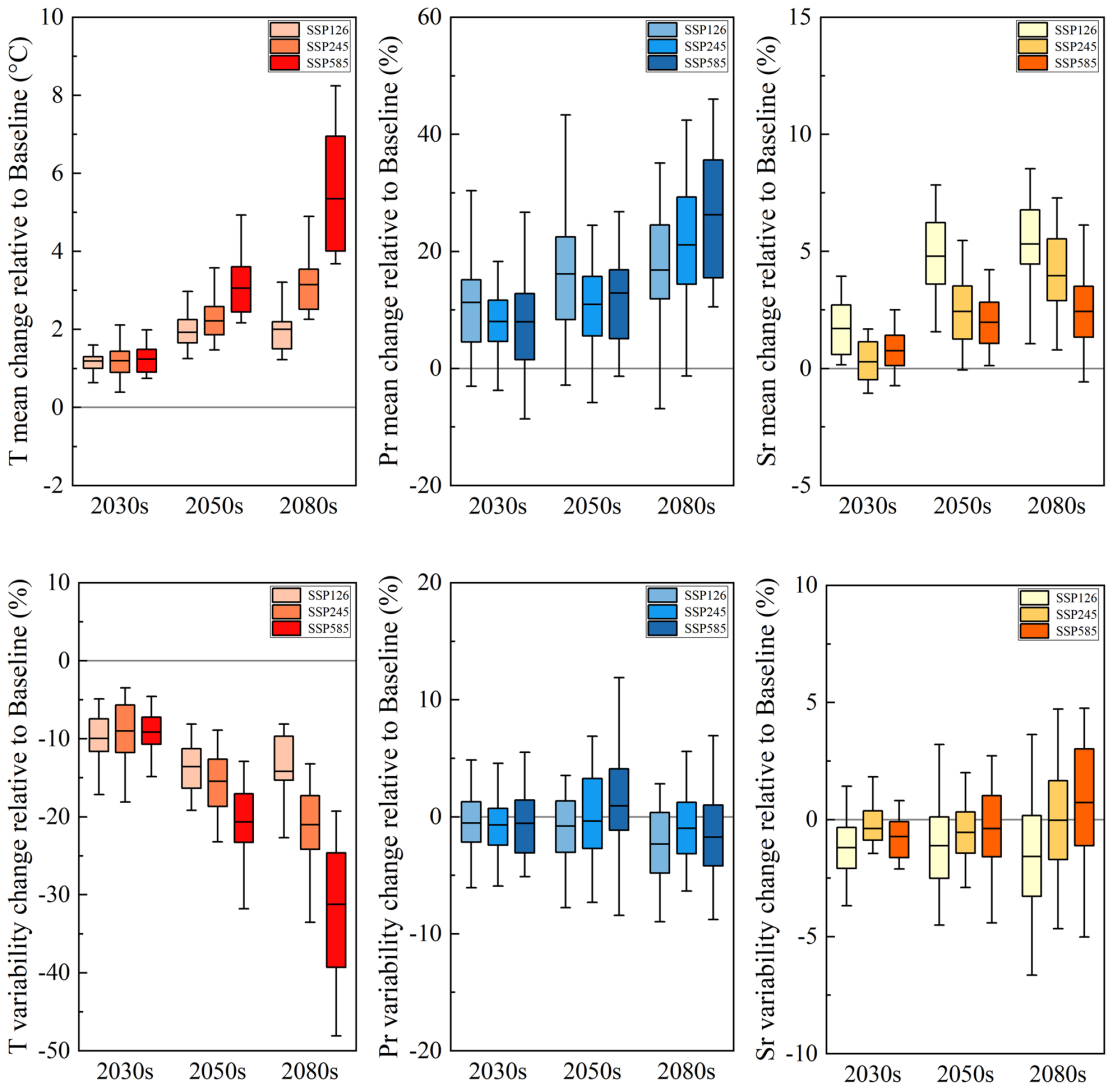
Changes in average temperature, total precipitation, and solar radiation during winter wheat growth period in the future multi-model 2030s, 2050s, and 2080s periods relative to the base period.

The changes in average temperature, precipitation, and solar radiation during the future winter wheat growth period in the North China Plain (NCP) compared to the baseline period (1987–2014) are shown in Fig. 3. Multi-model simulation results (Table 3) indicate that the average temperature is projected to increase by 1.2 °C to 5.3 °C compared to the baseline period, with the magnitude of warming gradually increasing under stronger climate forcing scenarios and reaching its maximum in the far-term 2080s. Total precipitation is projected to increase under all scenarios, with a range of 8.0–31.2%. Solar radiation is also expected to increase by 0.3–5.3%, with the increase gradually growing over time, particularly under the low climate forcing scenario SSP126, where the increase in solar radiation is the largest.

**Table 3:** Changes in future climate variables relative to the base period (multi-model averages).

Climate variables	Climate scenarios	2030s	2050s	2080s	Trends in fluctuation
Average temperature	SSP126	+ 1.2 °C	+ 1.9 °C	+ 2.0 °C	− 10.2% → − 13.6% → − 14.2%
	SSP245	+ 1.2 °C	+ 2.2 °C	+ 3.1 °C	− 9.0% → − 15.5% → − 21.0%
	SSP585	+ 1.2 °C	+ 3.1 °C	+ 5.3 °C	− 9.2% → − 20.6% → − 31.2%
Total precipitation	SSP126	+ 11.3%	+ 16.2%	+ 16.8%	− 0.5% → − 0.8% → − 2.3%
	SSP245	+ 8.0%	+ 11.0%	+ 21.1%	− 0.7% → − 0.4% → − 1.0%
	SSP585	+ 8.0%	+ 12.9%	+ 26.3%	− 0.5% → − 0.9% → − 1.7%
Solar radiation	SSP126	+ 1.7%	+ 4.8%	+ 5.3%	− 1.2% → − 1.1% → − 1.6%
	SSP245	+ 0.3%	+ 2.4%	+ 4.0%	− 0.4% → − 0.5% → − 0.1%
	SSP585	+ 0.8%	+ 2.0%	+ 2.4%	− 0.7% → − 0.4% → + 0.7%

“→”indicates the fluctuation of climate elements from the 2030s to the 2050s and 2080s.

Analysis using multi-model ensemble means of daily climate variables under future scenarios found that the changes in climate fluctuations showed different characteristics (Fig. S5). Temperature fluctuations overall decrease, with a reduction range of 9.0–31.2%, and the largest decrease occurs under the far-term SSP585 scenario. This result aligns with other studies showing a decreasing trend in temperature fluctuations during the winter and spring seasons in northern China^60^. The fluctuation in total precipitation shows a small decreasing trend of 0.4–2.3%. The fluctuation in solar radiation is relatively small (− 1.6% to 0.7%), showing a decreasing trend in the near-term and mid-term, while under the far-term medium and high forcing scenarios, solar radiation fluctuations exhibit an increasing trend.

From the perspective of regional station responses (Fig. S6), the temperature trends at the six stations are consistent, indicating a spatially homogeneous feature of future climate warming. However, the magnitude of precipitation changes shows a clear latitudinal gradient effect. The increase in precipitation is generally greater at higher latitude stations than at lower latitude stations, suggesting significant spatial differences in the impact of future climate change on regional precipitation. The increase in solar radiation decreases with increasing latitude, with larger increases observed at lower latitude stations. Additionally, the impact of climate fluctuations varies across stations, particularly in terms of precipitation and solar radiation fluctuations, with significant differences in responses between northern and southern stations. The regional heterogeneity of climate responses should be considered in the design of adaptation strategies, with optimized agricultural management measures tailored to the characteristics of different stations.

### Assessment of the impact of future climate change on winter wheat yield

**Fig. 4:**
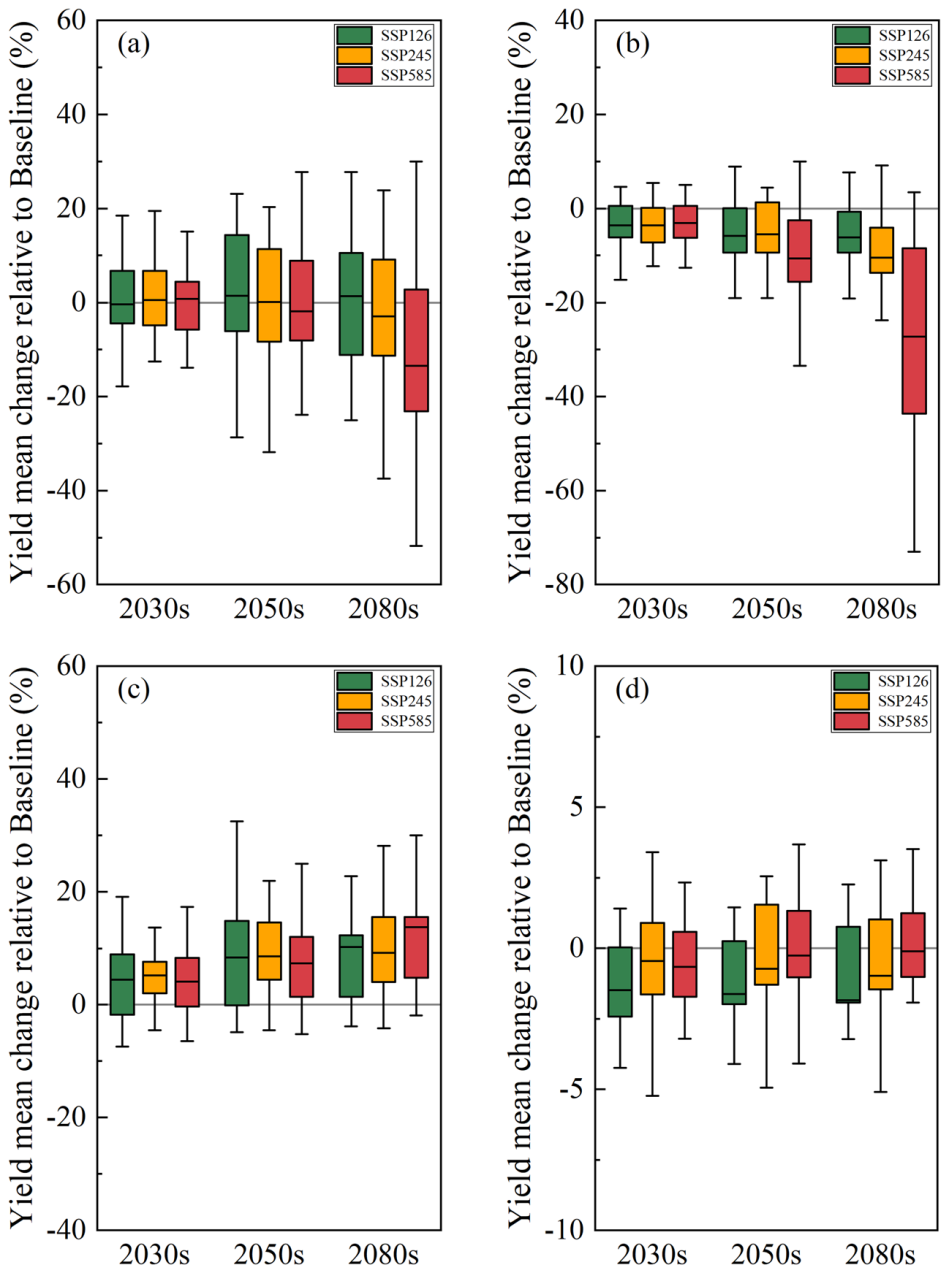
Impact of future changes in climate factors on winter wheat yield in the NCP. (**a**) Overall impact of climate change; (**b**) Impact of temperature change; (**c**) Impact of precipitation change; (**d**) Impact of radiation change.

Under the overall influence of future climate change, the simulated winter wheat yield in the North China Plain (NCP) showed a trend of increasing by 1.5% in the near-term 2030s and decreasing by 13.4% in the far-term 2080s (Fig. 4a). At the regional station level, the impact of future climate change on yield exhibited significant spatial heterogeneity. Under the low and medium climate forcing scenarios (SSP126 and SSP245), agricultural irrigation stations at higher latitudes generally showed an increase in yield, while those at lower latitudes showed a decrease in yield. The rainfed agricultural station Nanyang also exhibited an increase in yield. However, under the high climate forcing scenario (SSP585), agricultural irrigation stations at higher latitudes began to show a decrease in yield (Fig. S7). These results indicate that the impact of future climate change on winter wheat yield not only has distinct temporal characteristics but also exhibits regional differences, particularly under high forcing scenarios.

From the perspective of the relative impacts of future climate factors (temperature, precipitation, and radiation) on winter wheat yield (Fig. 4b–d), rising temperatures were the primary driver of yield reduction, with an average reduction of 8.4%. The degree of yield reduction intensified with increasing climate forcing scenarios, reaching an average reduction of 27.3% in the far-term 2080s. In contrast, precipitation changes mainly contributed to yield increases, with the magnitude of increase gradually growing over time, ranging from 4.1% to 13.7%. Radiation changes had a relatively minor impact, resulting in a slight yield reduction of 0.1–1.8%. The impacts of future climate factors on winter wheat yield varied across different scenarios and time periods (Table 3), clearly demonstrating that rising temperatures are the main driver of future yield reduction, while increased precipitation partially mitigates the negative effects of climate change.

**Fig. 5:**
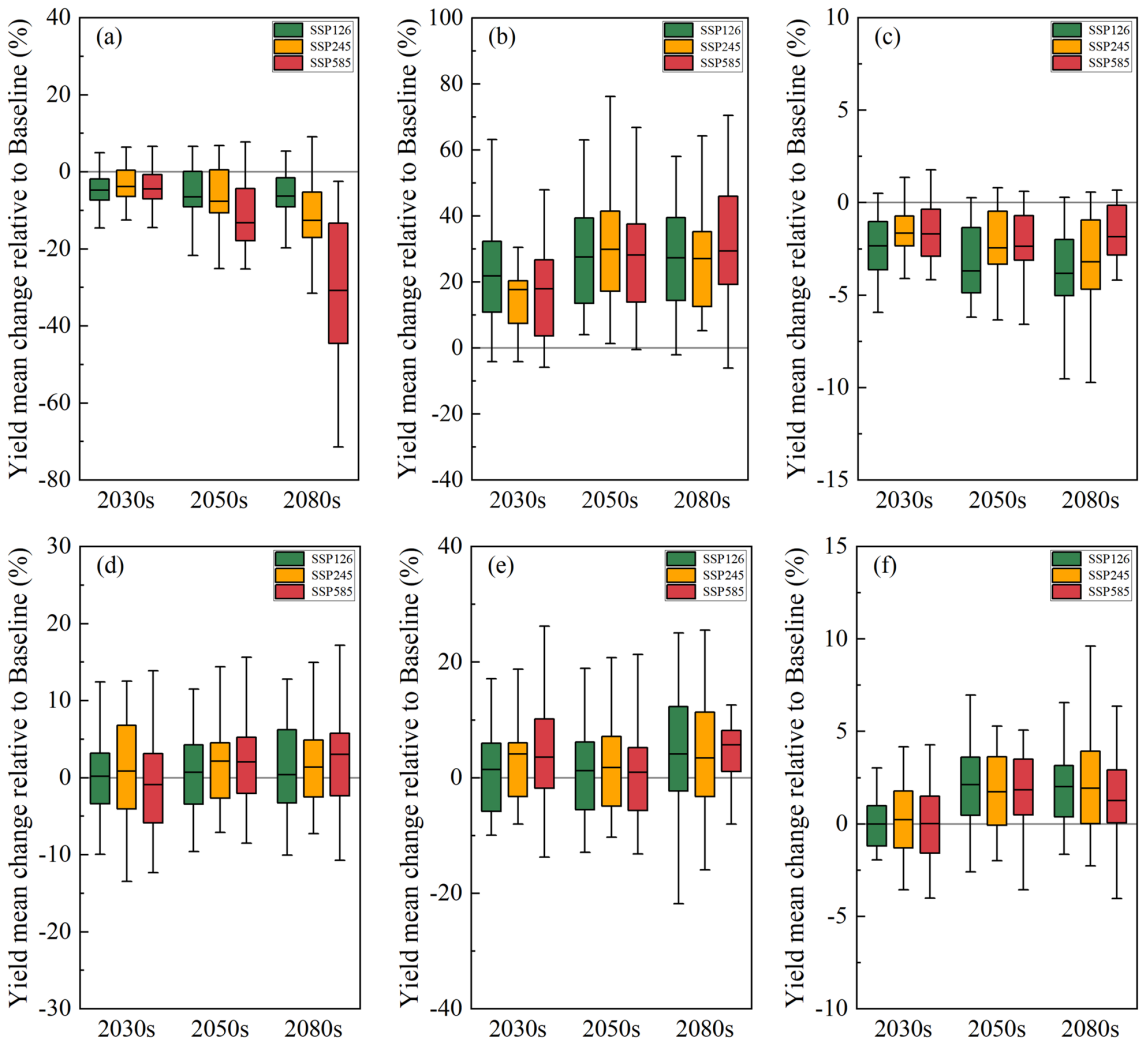
Impact of future changes in temperature, precipitation, and radiation trends and fluctuations on winter wheat yields. (**a**) Impact of temperature trends; (**b**) Impact of precipitation trends; (**c**) Impact of radiation trends; (**d**) Impact of temperature fluctuations; (**e**) Impact of precipitation fluctuations; (**f**) Impact of radiation fluctuations.

Regarding the impacts of climate trends and fluctuations on winter wheat yield (Fig. 5), climate trends had significant effects, with distinct differences among climate factors(Fig. 5a–c). Temperature trends led to an average yield reduction of 10%, consistent with the overall impact of temperature, and the degree of reduction increased with stronger climate forcing scenarios. Precipitation trends significantly promoted yield increases, with an average increase of 25%, and this effect was strengthened by interannual variations. Radiation trends resulted in a slight yield reduction of 3%, but the degree of reduction decreased with increasing climate forcing scenarios, showing a relatively smaller impact compared to other factors.

Additionally, simulation results showed that future fluctuations in temperature, precipitation, and radiation had minor effects on winter wheat yield (Fig. 5d–f). Temperature fluctuations led to a slight yield increase of 1.1%, while precipitation fluctuations mainly contributed to yield increases, with an average increase of 2.9%. Radiation fluctuations also resulted in a slight yield increase of 1.2%. Overall, compared to the dominant role of climate trends, the impacts of climate fluctuations on winter wheat yield were relatively small, all showing a slight increasing trend (Table 4).

**Table 4:** Differential impacts of climate trends and fluctuations.

Climate variable	Simulation scenario	2030s	2050s	2080s
Temperature	Overall impact	− 3.4%	− 7.3%	− 14.6%
	Trend impact	− 4.3%	− 9.1%	− 16.6%
	Variability impact	+ 0.1%	+ 1.6%	+ 1.6%
Precipitation	Overall impact	+ 4.6%	+ 8.1%	+ 11.1%
	Trend impact	+ 19.1%	+ 28.5%	+ 27.9%
	Variability impact	+ 3.1%	+ 1.3%	+ 4.4%
Solar radiation	Overall impact	− 0.9%	− 0.9%	− 1.0%
	Trend impact	− 1.9%	− 2.8%	− 2.9%
	Variability impact	+ 0.1%	+ 1.9%	+ 1.7%

The data in the table are the average impacts of climate variables under multi-model and multi-scenario scenarios.

### Analysis of the contribution of future climate change to winter wheat yield

**Fig. 6:**
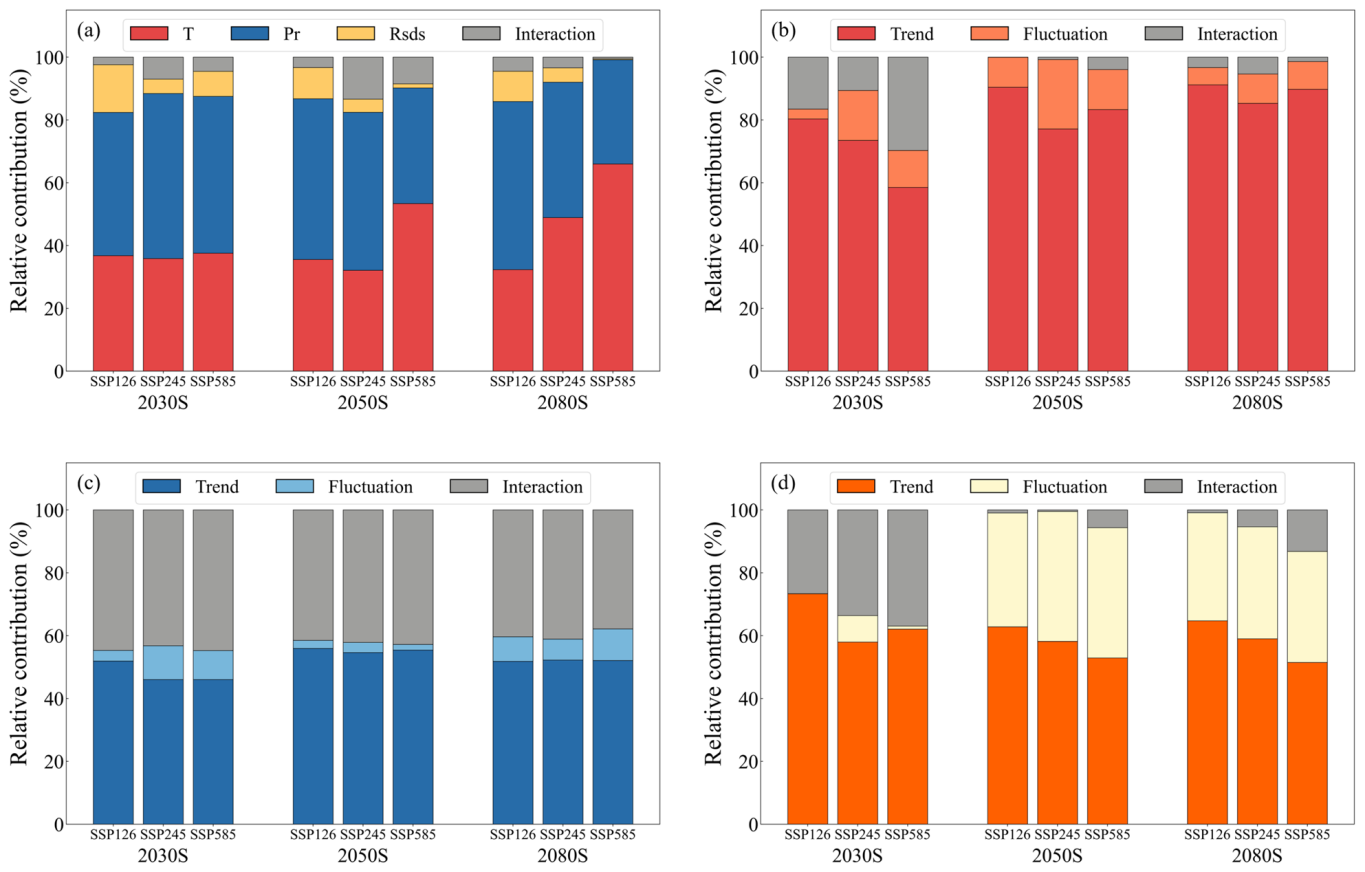
Contributions of temperature, precipitation, radiation, and interaction to the impact on winter wheat yield. (**a**) Relative contribution among climate elements; (**b**) Relative contribution to temperature trends and fluctuations; (**c**) Relative contribution to precipitation trends and fluctuations; (**d**) Relative contribution to radiation trends and fluctuations.

According to the contribution analysis of future temperature, precipitation, and radiation to winter wheat yield changes (Fig. 6a), the main climatic factors affecting future winter wheat yield changes in the North China Plain were temperature and precipitation, with average contributions of 42% and 46%, respectively. In the 2030s and the 2050s, precipitation had a significant impact on winter wheat yield. However, as time progressed from SSP126 to SSP585, the contribution of temperature gradually increased. In the 2080s, temperature had a more substantial impact on winter wheat yield, whereas the overall contribution of radiation was relatively small, with an average contribution of 7%; its impact diminished over time.

Based on the simulated contributions of temperature, precipitation, radiation trends, and fluctuations to winter wheat yield in the 2030s, 2050s, and the 2080s (Fig. 6b–d). The overall impact of temperature was primarily driven by temperature trends, with a maximum contribution. The average contribution rate of temperature trends to winter wheat yield was 81%, whereas the average contribution rates of temperature fluctuations and interactive effects were smaller, at 11% and 8%, respectively. The overall impact of precipitation was characterized by a greater contribution from precipitation trends than from precipitation fluctuations. The average contribution of precipitation trends to winter wheat yield was 52%, precipitation fluctuations contributed an average of 6%, and the interactive effects between precipitation trends and fluctuations were substantial at 42%. The overall impact of radiation was significantly influenced by radiation trends, with an average contribution rate of 60%. Radiation fluctuations contributed an average of 26% and their contribution rates gradually increased over time. The interactive effects between the radiation trends and fluctuations were relatively minor, with an average contribution rate of 14%.

### Exploration of management measures to address climate change

**Fig. 7:**
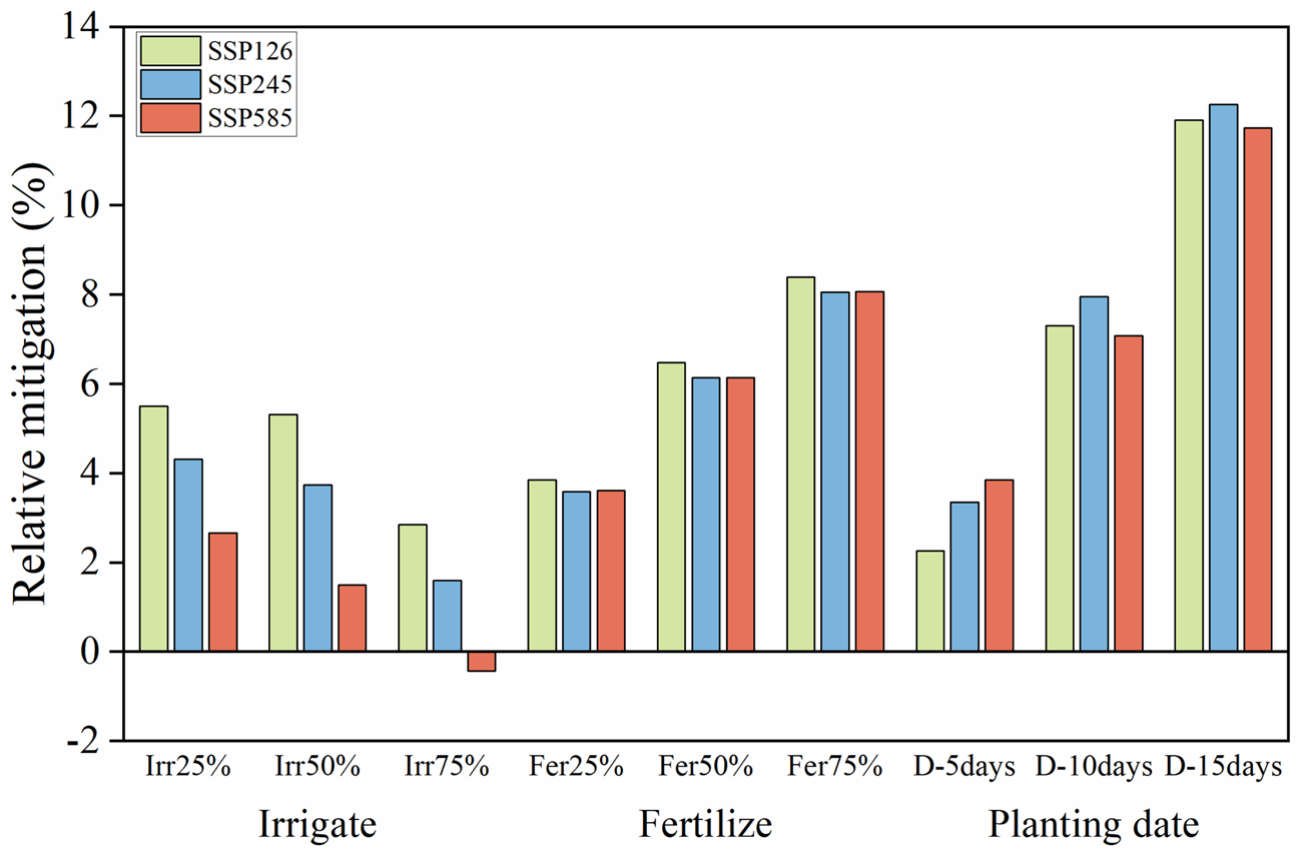
Mitigation effect of management measures on future climate-induced yield reduction.

Taking the agricultural irrigation stations of Liaocheng and Xinxiang in the southern part of the NCP, which are greatly affected by reduced production, as examples. The research findings suggest that, in the context of future climate change, the three management measures of irrigation, fertilization, and adjusting planting dates can mitigate the adverse effects of climate-induced yield reduction (Fig. 7), among which adjusting planting dates and increasing fertilization show more pronounced effects. On average, delaying planting dates mitigated a 7.5% reduction in yield, whereas increasing fertilization mitigated a 6% reduction. The impact of increasing the irrigation water was relatively small, with an average yield reduction of 3%. As the management level strengthened, the mitigation effects of adjusting planting dates and increasing fertilization gradually increased, whereas the mitigation effect of increasing irrigation water diminished with an increase in irrigation volume. Under SSP585, adjusting planting dates and increasing fertilization continued to effectively alleviate yield reductions, whereas the effectiveness of increasing irrigation water management tended to decline.

## Discussion

### Evaluation of the impact of climate change characteristics on winter wheat yield in the NCP

Our results indicate that the winter wheat yield in the NCP will shift from an increase in the near-term 2030s to a decrease in the far-term 2080s relative to the baseline period, with the magnitude of the decrease gradually increasing. At the site level, irrigated agricultural sites at higher latitudes showed increased yields, while those at lower latitudes exhibited decreased yields. Rainfed agriculture at the Nanyang site, located at a lower latitude, also showed increased yields.

Further analysis of the impact of climate change on winter wheat yield at regional stations (Fig. S8) revealed that future increases in temperature and changes in radiation would lead to a reduction in winter wheat production in the NCP. Rising future temperatures emerged as the primary factor causing this reduction, resulting in decreased winter wheat yields at all six study sites (except for the Liaocheng site under the SSP126 and SSP245 scenarios). Increased precipitation during the growing season played a crucial role in the potential boost of winter wheat yield in the NCP. Additional precipitation in the future contributed to increased winter wheat yields at all six study sites, with a more noticeable potential increase at the rainfed Nanyang site. The findings of this study are consistent with those of previous studies in various respects. For instance, Rosenzweig et al.^19^ found that global warming may cause wheat yield losses in low- and mid-latitude regions, which aligns with our observations in the NCP. Under the RCP2.6, RCP4.5, and RCP8.5 climate change scenarios, Yang et al.^51^ projected that the probability of reduced irrigated wheat yield in China would steadily increase, with winter wheat yield reductions of 2%, 6%, and 9%, respectively, by the end of the century, while rainfed wheat yields would increase by more than 21%, 22%, and 25%, respectively. These findings further support the negative impact of global warming on wheat yields, particularly in irrigated agricultural regions.

Regarding climate fluctuations, it was found that the weakening of future temperature, precipitation, and radiation fluctuations has the potential to increase winter wheat yield. However, their impact is weaker than that of climate trends. Other studies have indicated that fluctuations in climatic factors are an unstable manifestation of climate change. Increased temperature and precipitation fluctuations have negative effects on wheat yield in Nepal^61^, while climate fluctuations are a major driver of crop yield reduction in some northern regions of China, with temperature fluctuations affecting wheat yield by approximately 4% and precipitation fluctuations affecting yield by approximately 2%^31^.

It was also discovered that the six agrometeorological stations in the region had different degrees of response to future changes in different climatic factors (Fig. S9). By analyzing the degree of contribution of different climatic factors to winter wheat yield, it was found that the winter wheat yield at agricultural irrigation stations at higher latitudes was mainly affected by precipitation. The potential yield increase effect exceeded the potential yield reduction effect caused by temperature and radiation and finally showed the characteristics of yield increase. However, the winter wheat yield at agricultural irrigation stations at lower latitudes was mainly controlled by temperature factors, and the potential impact of temperature increase was greater, resulting in a reduction in yield. The rain-fed agricultural station in Nanyang at lower latitudes was more sensitive to precipitation changes and was greatly affected by precipitation, and its yield increase effect was more pronounced. The finding that winter wheat in regions with low growing season temperatures and regions with high growing season temperatures have different sensitivity to temperature rise, which is also consistent with the finding that climate warming reduces wheat yield in regions with high temperatures^62,63^.

### Analysis of field management measures in response to climate change

Different management measures have varying capacities for mitigating the impacts of climate change. The results indicate that appropriately delaying the winter wheat planting date and increasing field fertility can effectively alleviate the adverse effects of future temperature trends on winter wheat yield in the southern irrigation areas of the NCP, whereas the mitigation effect of increased irrigation is relatively weak.

Further exploration of the differential mitigation capacities of various management measures revealed that climate warming plays a guiding role in adjusting crop planting dates. The optimal temperature conditions for winter wheat sowing range from 15 to 17 °C^64^, and related research on suitable temperatures provides a scientific basis for optimizing sowing strategies. Due to the projected increase in temperature during the winter wheat sowing period in the NCP, the optimal sowing time has shifted (Fig. S10). Compared to the historical baseline period, under the SSP126 low-forcing climate scenario, the suitable sowing time is delayed by 7 days; under the SSP245 medium-forcing scenario, it is delayed by 10 days; and under the SSP585 high-forcing scenario, it is delayed by 14 days. The overall temperature increase during the early growth stage shortens the emergence time of winter wheat, leading to excessive nutrient consumption, affecting physiological metabolism, and resulting in insufficient overwintering capacity, which subsequently impacts growth and development in later stages. Appropriately delaying the planting date may mitigate the damage caused by high temperatures during the growth period and even increase yield. Studies have also shown that rising winter temperatures in the NCP can delay the wheat sowing period, increasing total wheat yield by 4–6%^65^.

Increasing fertility is another important measure for adapting to climate change. Rational fertilization based on water availability and using water to promote fertilization are key technologies for promoting crop growth and improving yields in comprehensive agricultural development^66,67^. Particularly in the context of increased precipitation during the future growth period, higher moisture levels facilitate the dissolution of nutrient elements in the soil, improving the absorption and utilization efficiency of field fertilizers by winter wheat. Similar trends have been observed in the long-term combined application of organic and inorganic fertilizers on wheat yield^68^. A higher water-fertilizer coupling effect can mitigate the negative effects of excessive nutrient consumption caused by rising temperatures.

The limited effectiveness of increased irrigation measures may be attributed to the fact that existing management practices have already partially mitigated the potential yield reduction caused by future temperature increases. By analyzing the natural water deficit of winter wheat during the growing season in the NCP (Fig. S11), it was found that the natural water deficit under the SSP585 high-forcing scenario is lower than that under the SSP126 and SSP245 scenarios, and the natural water deficit gradually decreases over time. Excessive increases in water management levels may not result in significant improvements, especially when over-irrigation exacerbates yield reduction, which is consistent with our findings on the limited mitigation capacity of irrigation management practices. Under conditions of increased precipitation during the future winter wheat growing period, excessive irrigation may reach an upper limit in mitigating the cooling effect of high temperatures, and excessive moisture may lead to reduced winter wheat yields.

### Response of varietal differences to climate change and adaptation strategies

Varietal differences have a significant impact on the mechanisms by which winter wheat yield responds to climate change^69^, but their role in regional adaptability requires further clarification. To this end, this study focused on the key roles of varietal parameters (e.g., photoperiod sensitivity P1D, grain filling rate G2) and soil properties (e.g., hydraulic conductivity, field capacity) in the response of winter wheat yield to climate change, based on the DSSAT model. The results indicate that differences in varietal parameters influence the adaptability of winter wheat to climate change. For example, higher P1D values reflect a variety’s dependence on long photoperiods but may lead to yield reduction under rising temperatures due to shortened growth stages, while lower G2 values can result in insufficient grain filling under high-temperature conditions, affecting yield. Additionally, differences in soil hydraulic conductivity and field capacity regulate water use efficiency, with higher field capacity effectively mitigating the negative effects of precipitation fluctuations on yield, while lower hydraulic conductivity may limit water infiltration.

Regional analysis showed that the Bazhou station in the north exhibited significant yield reduction under increasing temperatures due to higher P1D and lower G2 values, while the Nanyang station in the south showed an increasing yield trend under rainfed conditions due to higher precipitation utilization efficiency and stronger temperature adaptability. These findings are consistent with previous studies^70,71^, further validating the critical role of varietal parameters and soil properties in the response of winter wheat yield.

Based on these findings, adaptation strategies need to be tailored to specific conditions. For example, in the north, breeding low-P1D varieties (< 80 °C·d) can alleviate photoperiod limitations, and optimizing soil structure can balance hydraulic conductivity and water retention. In the south, improving the grain filling rate (G2 > 50 mg m⁻^2^ d⁻^1^) and enhancing soil hydraulic conductivity through organic matter amendments are recommended. Additionally, the broad adaptability of varieties is of great significance to breeders. Future research should further explore adaptation strategies for different varieties to address the uncertainties posed by climate change. For instance, breeding more stress-resistant varieties can effectively improve the yield stability of winter wheat.

### Response suggestions

This research revealed that winter wheat in the NCP was significantly affected by future temperature trends, emphasizing the importance of winter wheat planting to mitigate the threat of high temperature and heat damage in the future. This study indicates that appropriately delaying the winter wheat planting date and increasing fertilization levels can effectively alleviate the adverse effects of future temperature increases, although the impact of increased irrigation management is limited. This finding suggests that the current level of agricultural management measures require further improvement. Future efforts should focus on varietal improvements, introducing heat-tolerant varieties to adapt to higher temperatures, or implementing other technological advancements to stabilize the length of the growth period, prevent its shortening, and address the potential impacts of climate change.

Under future climatic conditions, appropriate water-saving management measures can optimize the utilization of agricultural water resources in the region. With increasing population and economic development in the North China Plain, a key grain-producing area, the demand for irrigation water in agriculture has risen significantly, resulting in an escalating imbalance between water supply and demand. The threat of water scarcity in this region has become increasingly severe ^72^. Due to the projected increase in precipitation during the future growing season in the North China Plain, crop water demand may not be a limiting factor for crop growth. Precipitation could potentially contribute to increased yields of winter wheat. Exploring the adoption of minimal irrigation strategies, enhancing water use efficiency, and developing water-saving varieties could be effective solutions for the future development of agricultural irrigation areas under changing climatic conditions.

### Limitations and future prospects

This study quantitatively simulated the impact of climate trends and variability on winter wheat yields by constructing future climate trends and climate fluctuation scenarios and using crop models. It provides differentiated insights through a comprehensive assessment of the contribution levels of various climate change factors to crop yields and an exploration of the adaptive and mitigating capabilities of different agricultural management measures to climate change. In contrast to previous research, this study combines the analysis of climate trends and climate variability, comprehensively investigates the dominant climatic factors affecting irrigated and rain-fed agriculture, and proposes optimization recommendations for regional agricultural climate adaptation strategies.

This study had some limitations. It employs a single crop growth model, and the results from a single model may be influenced by the model’s sensitivity to various climatic factors, leading to biased outcomes. The use of multiple crop-growth models to investigate the impact of future climate change on winter wheat yields could enhance the accuracy of the results. However, the DSSAT-CERES wheat model selected in this study is commonly used for regional crop assessments and demonstrates high frequency and accuracy. Its application to the North China Plain may outperform other models^73,74^. Furthermore, this study did not separately discuss the impact of different extreme weather events on winter wheat yields. Instead, it includes extremes related to climatic fluctuations. Individual extreme weather events can also lead to yield reduction. Future research should isolate the influence of extreme weather events from climate fluctuation for more targeted management.

Furthermore, this study did not consider the impacts of dynamic changes in crop varieties, management technology levels, or the effects of preceding crops (e.g., summer maize) when simulating the impact of future climate change on winter wheat yields. Preceding crops have a significant influence on soil moisture, planting dates, and other factors, especially under rainfed conditions, where their impact may intensify water competition, thereby affecting the growth cycle and final yield of winter wheat. Therefore, future research could incorporate the effects of preceding crops into the model to assess their potential impact on wheat production and explore how to mitigate these adverse effects through reasonable crop rotation, tillage practices, and water management measures. While the study results align well with existing research conclusions, there may still be some differences from actual future scenarios. Subsequent research should consider dynamic changes in future crop varieties and field management practices when simulating the impacts of future climate change.

Future studies could incorporate atmospheric carbon dioxide (CO₂) concentrations to reduce uncertainties in simulating climate impacts. Current research suggests that increasing CO₂ levels may mitigate the adverse effects of warming on wheat yields^75,76^. In this study, by setting CO₂ concentration levels under three future emission scenarios, similar results were obtained. With increasing CO₂ concentrations, future wheat yields showed an increasing trend (Fig. S12) and the average contribution rate of CO₂ to winter wheat yield was 26% (Fig. S13). However, under the three long-term emission scenarios, the yield-increasing effect was constrained, and the contribution rate of CO₂ gradually decreased (Fig. S13), consistent with the analysis of the potential impacts of climate change. Subsequent research could consider incorporating atmospheric CO₂ concentrations and certain air pollutants into the study.

## Conclusion

This study quantitatively assessed the impacts of climate change on winter wheat yields in the North China Plain (NCP) by integrating future climate trend and fluctuation scenarios with the DSSAT crop growth model. The results indicate that winter wheat yields in the NCP will transition from an initial increase to a significant decline by the end of the century, with projected yield losses reaching 13.4% in the 2080s. Rising temperatures are identified as the primary driver of yield reduction, causing an average potential yield loss of 8.4%, while increased precipitation partially offsets this negative effect, contributing to potential yield enhancements.

The impacts of climate change differ markedly between irrigated and rainfed agricultural systems. In northern regions, irrigated agriculture exhibits yield increases due to temperature rise, whereas southern regions experience yield declines under the same warming conditions. Rainfed agriculture, however, demonstrates greater sensitivity to precipitation changes, with increased rainfall driving yield improvements. Notably, low-latitude irrigated areas suffer more severe yield losses from temperature rise, with potential reductions of up to 17%.

To address future climate risks, adaptive strategies such as appropriately delaying winter wheat sowing dates (to mitigate heat stress) and enhancing field fertility are recommended to alleviate temperature-induced yield losses. Additionally, optimizing irrigation management and leveraging climate resources could help resolve regional water resource constraints, offering sustainable pathways for agricultural development in the NCP.

## Electronic supplementary material

Below is the link to the electronic supplementary material.

Supplementary Material 1

## Data Availability

The data that support the findings of this study are available from the corresponding author upon reasonable request.
