# Supplementary material for: Impact of future climate trend and fluctuation on winter wheat yield in the North China Plain and adaptation strategies
Source: Sci Rep. 2025 Jul 1;15:21882. doi: 10.1038/s41598-025-06370-6 (PMC12218318; doi:10.1038/s41598-025-06370-6)
Supplement: Supplementary file 1 — Supplementary Material 1 [file 41598_2025_6370_MOESM1_ESM.docx]

**Supplementary Materials**

**Impact of future climate trend and fluctuation on winter wheat yield in the North China Plain and adaptation strategies**

Jinpeng Hu^1,2^, Yichen Li^4^, Peijun Shi^1,2,3*^

1 State Key Laboratory of Earth Surface Processes and Disaster Risk Reduction (ESPDRR), Beijing 100875, China

2 Key Laboratory of Environmental Change and Natural Disasters of Chinese Ministry of Education, Beijing Normal University, Beijing 100875, China

3 College of Arts and Sciences, Beijing Normal University, Zhuhai 519087, China

4 College of Safety and Environmental Engineering, Shandong University of Science and Technology, Qingdao 266590, China

***Corresponding author:**

Peijun Shi, Beijing Normal University, Beijing 100875, China. Email: [spj@bnu.edu.cn](mailto:spj@bnu.edu.cn)

**Figure S1.** Spatial distribution of climatic elements of NCP during the historical period (1987-2014)


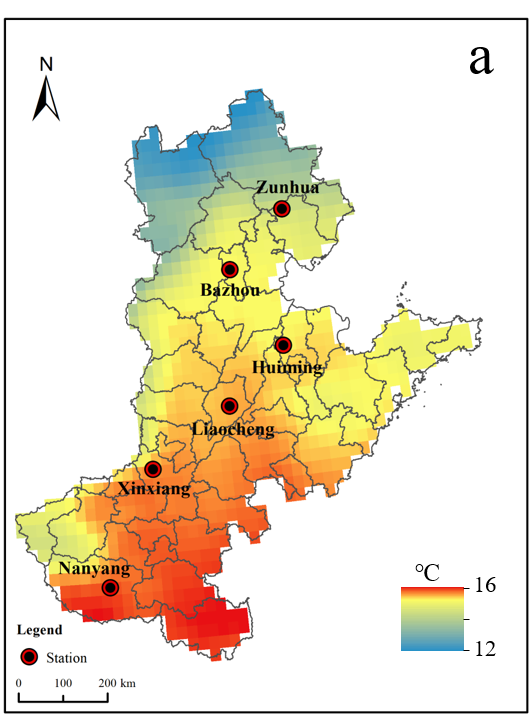

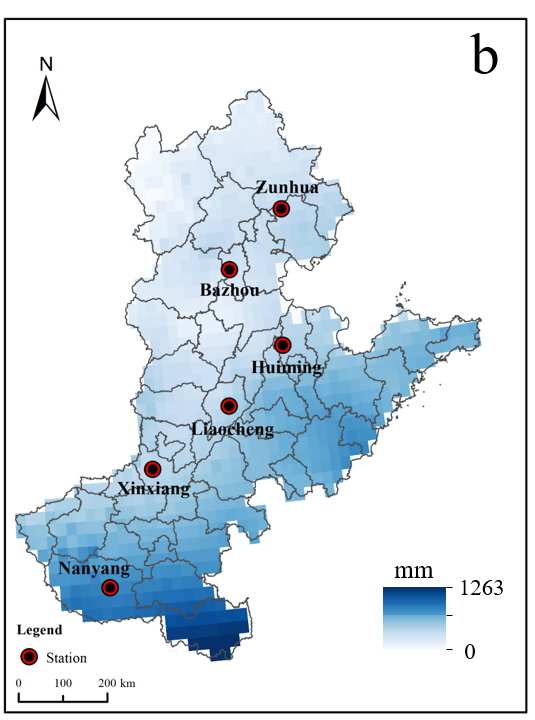

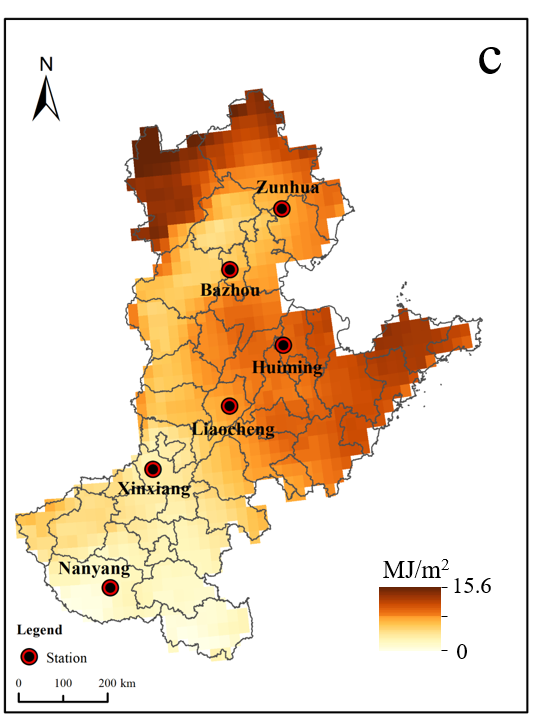


* a: Multi-year average monthly temperature; b: Annual mean precipitation; c: Annual average daily solar radiation. (Meteorological data provided by China Meteorological Data Service Center: http://data.cma.cn)

**Table S1** Soil profile parameters by station

| Parameter Name | Zunhua | Bazhou | Huimin | Liaocheng | Xinxiang | Nanyang |
| --- | --- | --- | --- | --- | --- | --- |
| Drainage coefficient | 0.3 | 0.3 | 0.3 | 0.3 | 0.3 | 0.3 |
| Runoff coefficient | 85 | 85 | 85 | 85 | 85 | 85 |
| Saturated water content (cm³/cm³) | 0.49 | 0.46 | 0.45 | 0.46 | 0.47 | 0.47 |
| Saturated hydraulic conductivity (cm/h) | 3.39 | 0.63 | 4.86 | 2.08 | 1.08 | 0.97 |
| Wilting point (cm³/cm³) | 0.11 | 0.12 | 0.07 | 0.12 | 0.13 | 0.14 |
| Field capacity (cm³/cm³) | 0.24 | 0.26 | 0.17 | 0.25 | 0.27 | 0.28 |
| Root growth coefficient | 1 | 1 | 1 | 1 | 1 | 1 |
| Bulk density (g/cm³) | 1.35 | 1.35 | 1.35 | 1.35 | 1.35 | 1.35 |

* Model inputs utilize soil parameters from the surface layer (0–30 cm).

**Table S2** Field management practices and input parameters by station

| Station Name | Planting Date | Sowing Rate(kg/ha) | Total Base Irrigation (mm) | Total Base Fertilization (kg/ha) |
| --- | --- | --- | --- | --- |
| Zunhua | 25 Sep | 219 | 180 | 193 N / 106 P |
| Bazhou | 3 Oct | 367 | 180 | 298 N / 206 P |
| Huimin | 6 Oct | 233 | 150 | 278 N / 323 P |
| Liaocheng | 11 Oct | 171 | 160 | 90 N / 90 P |
| Xinxiang | 11 Oct | 237 | 180 | 93 N / 117 P |
| Nanyang | 20 Oct | 230 | -- | 120 N / 120 P |

* N : nitrogen fertilizer, P: phosphorus fertilizer, “--": no input data.

**Table S3** RMSE between NEX-GDDP-CMIP6 (before and after bias correction) and observed climate data were effectively reduced after bias correction (mean value of 6 stations)

| Climate factors | *Pr* (mm) | | *SR* (MJ/m^2^) | | *T_max_* (°C) | | *T_min_* (°C) | |
| --- | --- | --- | --- | --- | --- | --- | --- | --- |
| Bias correction | Before | After | Before | After | Before | After | Before | After |
| GFDL-ESM4 | 8.74 | 8.54 | 6.93 | 5.60 | 5.69 | 5.65 | 4.38 | 4.32 |
| IPSL-CM6A-LR | 8.43 | 8.28 | 6.89 | 5.51 | 5.58 | 5.52 | 4.10 | 4.04 |
| MPI-ESM1 | 8.59 | 8.56 | 6.80 | 5.54 | 5.58 | 5.56 | 4.27 | 4.22 |
| MRI -ESM2 | 8.67 | 8.58 | 6.91 | 5.60 | 5.63 | 5.59 | 4.47 | 4.42 |
| UKESM1 | 8.42 | 8.41 | 6.81 | 5.47 | 5.62 | 5.49 | 4.51 | 4.36 |

* *Pr*: precipitation, *SR*: solar radiation, *T_max_*: maximum temperature, *T_min_*: minimum temperature.

**Table S4** Cultivars and planting years at study stations

| Station Name | Cultivar Name | Planting Years |
| --- | --- | --- |
| Zunhua | Dongle 10 | 2001, 2003, 2004 |
| Bazhou | Jingdong 8 | 2001, 2007-2009 |
| Huimin | Lumai 23 | 2001-2002, 2005-2009 |
| Liaocheng | Youmai 3 | 2003-2005 |
| Xinxiang | Zhoumai 9 | 1995-1997 |
| Nanyang | Yumai 18 | 1995, 1997, 2003, 2005 |

* The data in the table are the cultivars and planting years in which the research station participated in the validation.

**Figure S2.** Flowchart of the delta method (mean-state-adjusted bias correction) for climate data


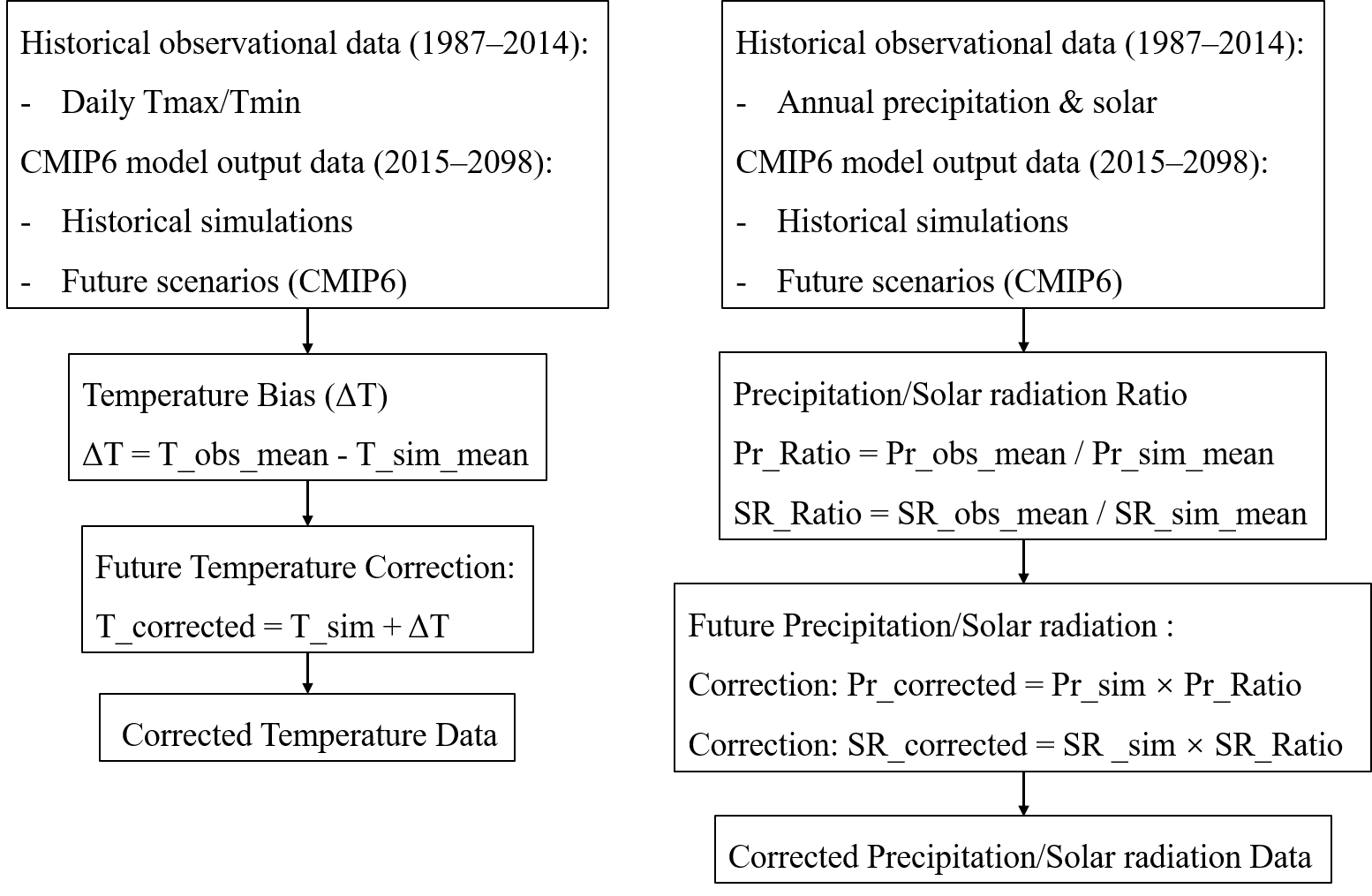


* This method calculates biases between historical observations and model simulations, then applies these biases to future scenarios to remove systematic errors.

**Figure S3.** Historical changes in water resources and agricultural fertilizer application on cropland in the North China Plain


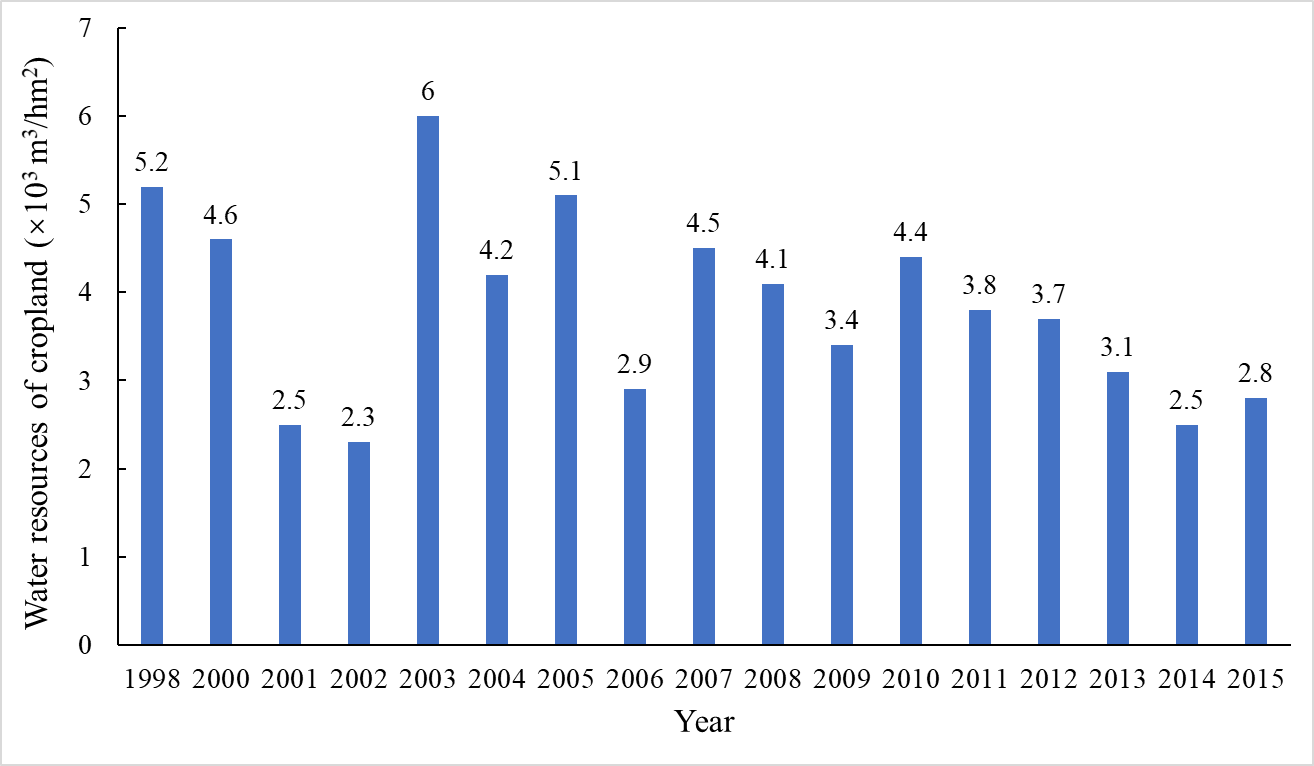

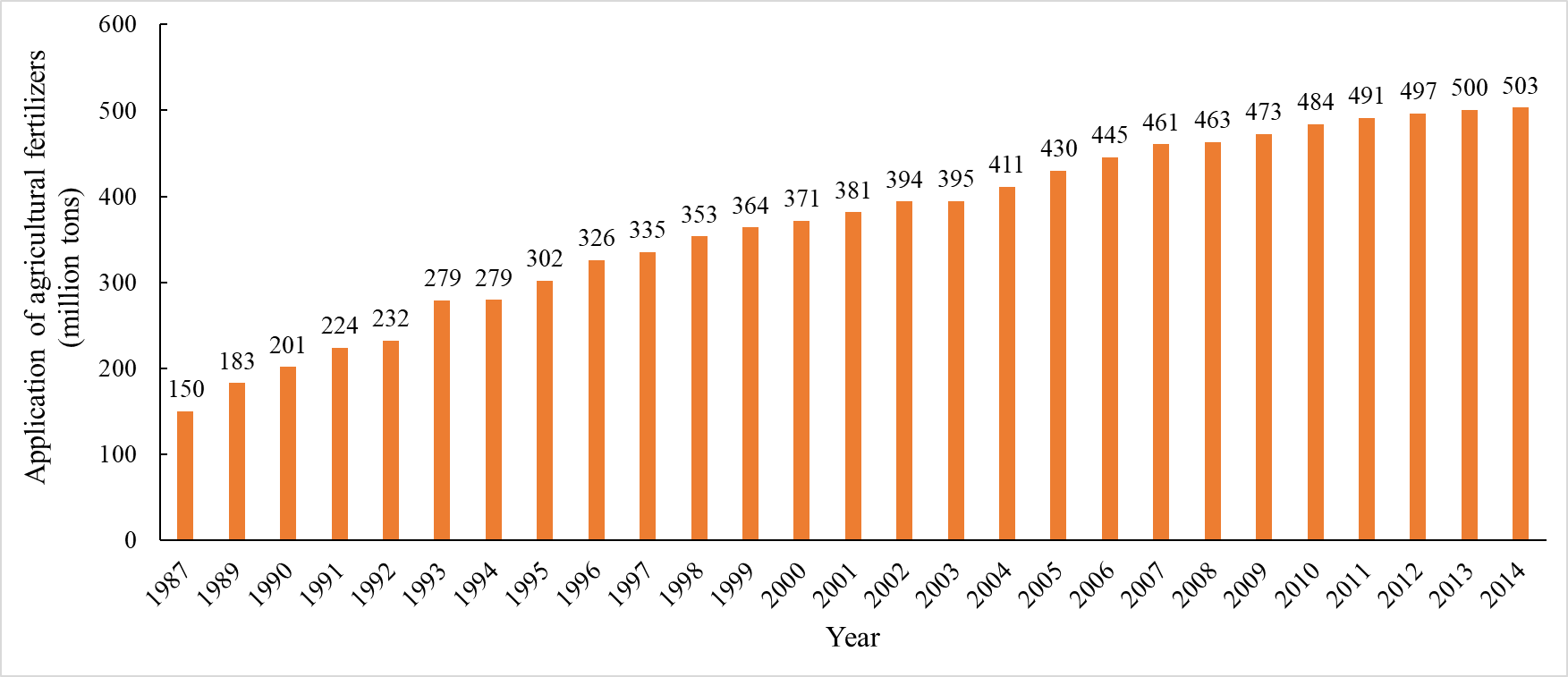


* Data on the average water resources per unit of cultivated land and agricultural fertilizer application amount in the North China Plain region are sourced from the China Statistical Yearbook.

**Table S5** The variety parameters of the site model were studied

| Station | Wheat variety | Variety parameter | | | | | | |
| --- | --- | --- | --- | --- | --- | --- | --- | --- |
|  |  | P1V | P1D | P5 | G1 | G2 | G3 | PHINT |
| Zunhua | Dongle 10 | 4 | 85 | 600 | 18 | 69 | 3.2 | 115 |
| Bazhou | Jingdong 8 | 6 | 118 | 600 | 34 | 22 | 3.18 | 37 |
| Huimin | Lumai 23 | 27 | 80 | 600 | 40 | 70 | 0.72 | 117 |
| Liaocheng | Yumai 3 | 60 | 45 | 600 | 26 | 35 | 0.7 | 53 |
| Xinxiang | Zhoumai 9 | 49 | 46 | 600 | 50 | 71 | 0.69 | 69 |
| Nanyang | Yumai 18 | 5 | 82 | 600 | 42 | 34 | 0.67 | 101 |

**Table S6** Yield stability at agricultural sites

| Site | CV（%） | Agricultural Type |
| --- | --- | --- |
| Zunhua | 15.8 | Irrigation |
| Bazhou | 15.4 | Irrigation |
| Huimin | 11.9 | Irrigation |
| Liaocheng | 10.1 | Irrigation |
| Xinxiang | 9.9 | Irrigation |
| Nanyang | 16.2 | Rainfed |

**Figure S4.** Validation of simulated and observed values for flowering date, maturity date and wheat yield


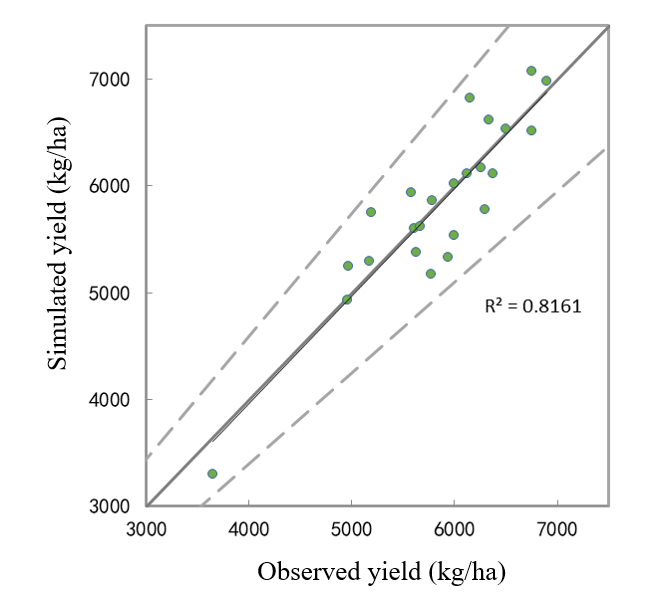

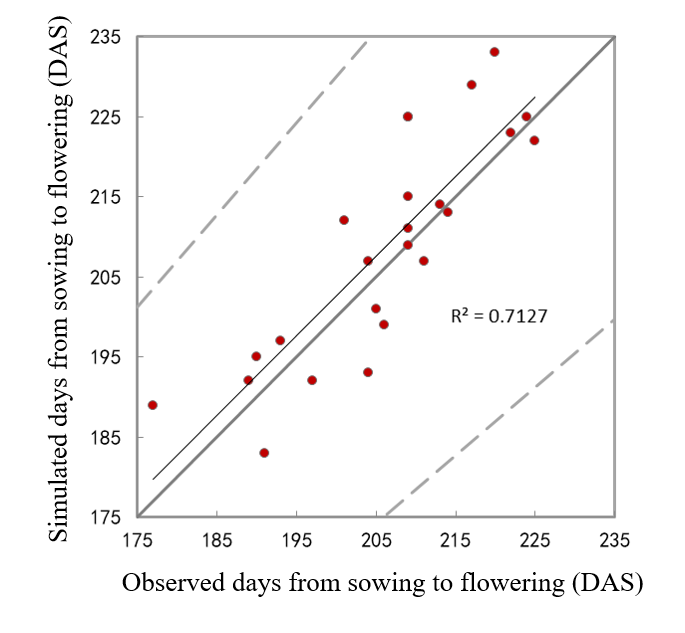

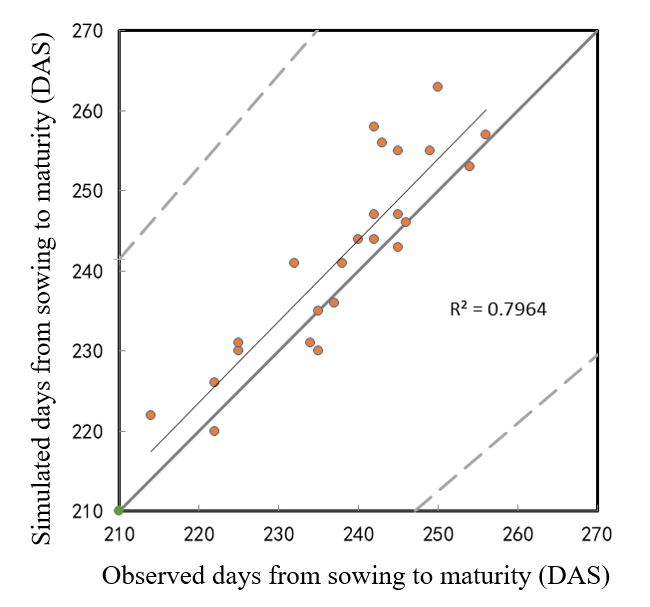


* DAS: days after sowing date, the solid line is the 1:1 line and the dotted line is the 15% prediction error line.

**Figure S5.** Projected daily changes in climate variables from multi-model ensembles


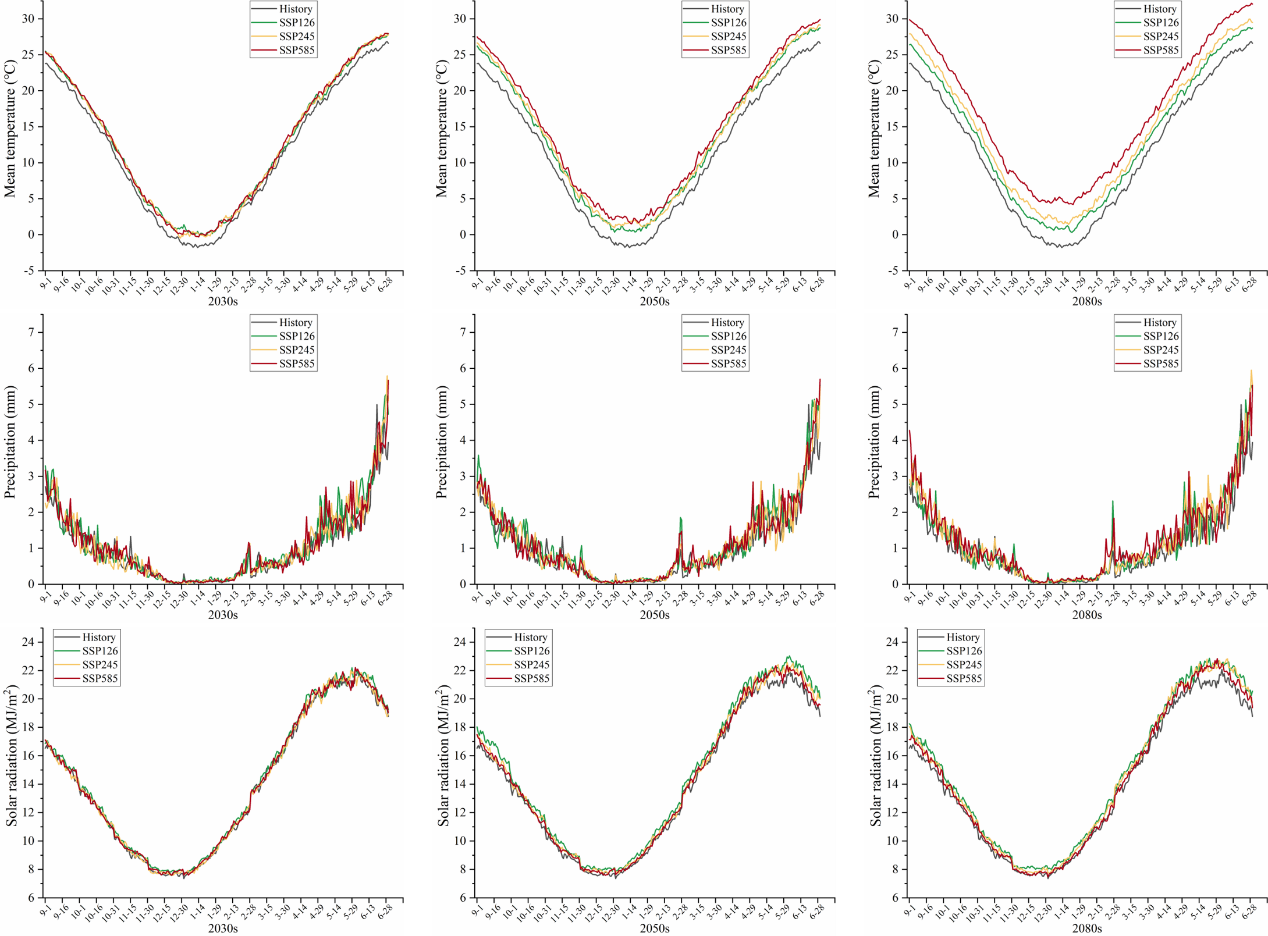


* A multi-model averaging approach is used to characterize the day-by-day variability of meteorological elements under future scenarios.

**Figure S6** Changes in climate trends and fluctuations during the future winter wheat growth period at regional sites

















* Each box plot includes a total of 15 results from five GCM models across three future periods.

**Figure S7.** Impact of future climate change on winter wheat yields at regional sites





* Each box plot includes a total of 15 results from five GCM models across three future periods.

**Figure S8.** Impact of future changes in climatic factors on winter wheat yields at regional sites








* a. Impact of temperature changes, b. Impact of precipitation changes, c. Impact of radiation changes（Each box plot includes a total of 15 results from five GCM models across three future periods）

**Figure S9.** Contributions of future temperature, precipitation, radiation, and interaction effects on winter wheat yields at regional sites





**Figure S10.** Changes in average temperature during the future winter wheat sowing period





* The temperature conditions suitable for sowing winter wheat are 15~17℃, the yellow area in the Fig is the suitable time range for sowing

**Figure S11.** Changes of natural water deficit during the future winter wheat growing season


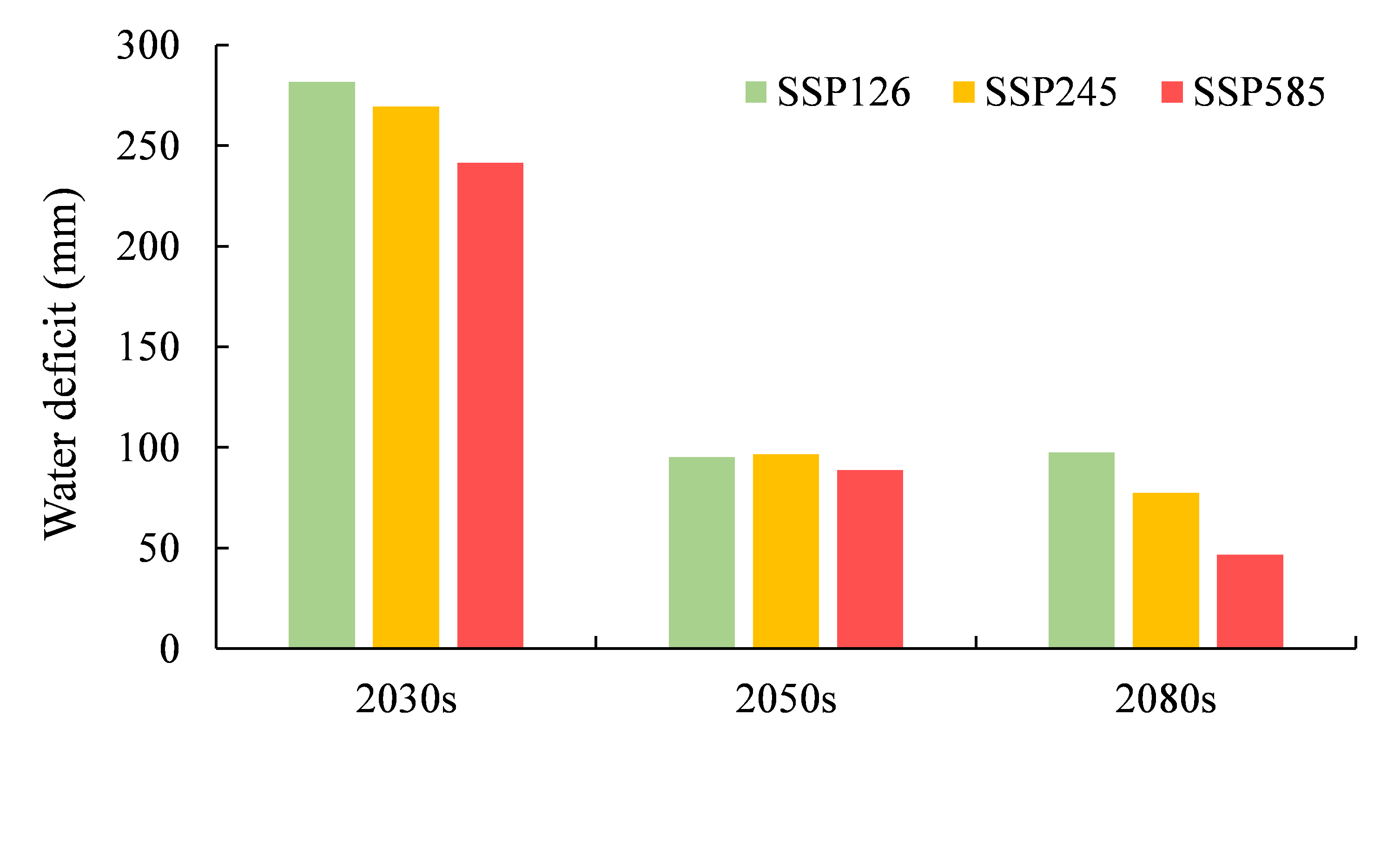


* According to the principle of farmland water balance, the natural water deficit of winter wheat can be described as the difference between winter wheat water demand and natural precipitation, in which winter wheat water demand is calculated by using the Penman-Monteith formula recommended by FAO.


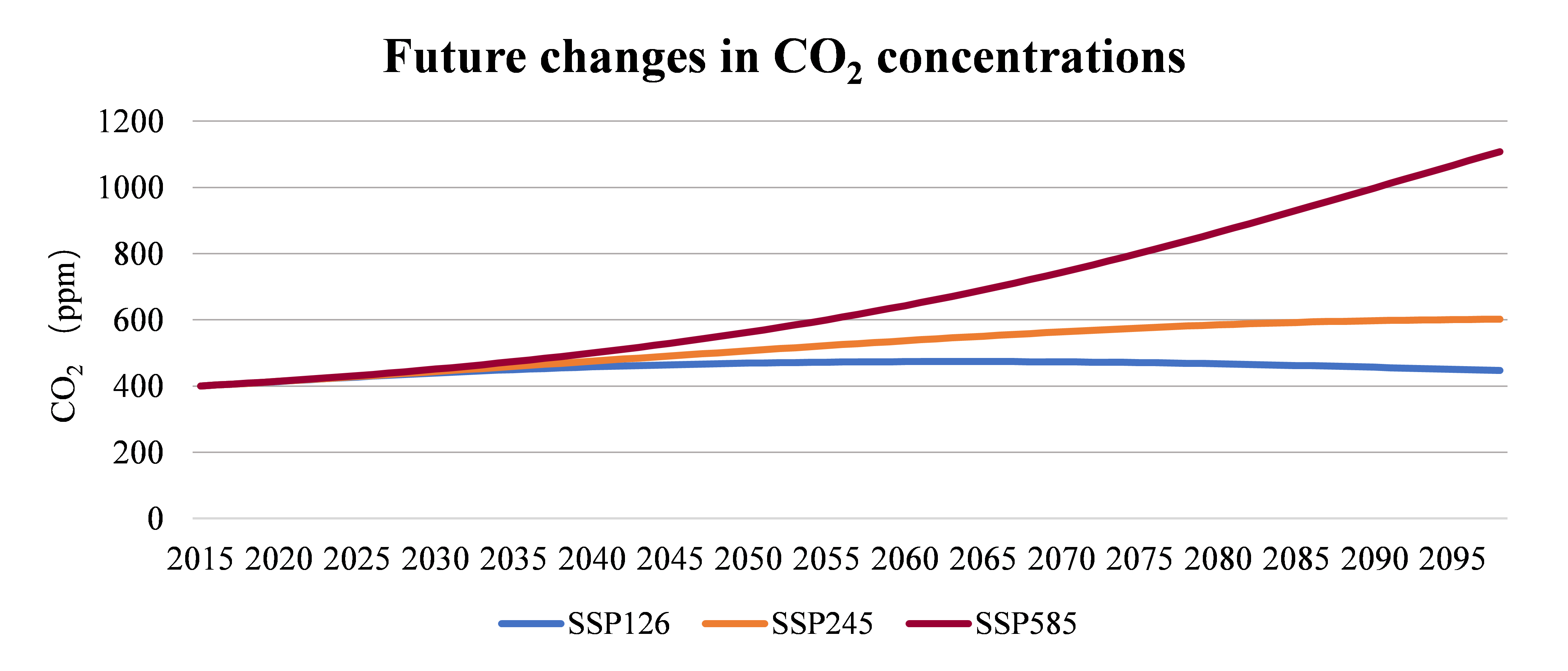


**Figure S12.** Effects of future CO_2_ increases on wheat yield

* Crop yield simulation using the average of CO_2_ concentrations under the corresponding scenarios of the future SSP126, SSP245, and SSP585 as a setup. (Carbon dioxide data source: Greenhouse Gas Factsheets, http://greenhousegases.science.unimelb.edu.au/#! /view)

**Figure S13.** Contribution of future CO_2_ impacts on winter wheat yield





* The figure shows the relative contributions of temperature, precipitation, radiation, carbon dioxide, and the interaction of the four factors to the effect of winter wheat yield.
